# Salt Effects on Caffeine across Concentration Regimes

**DOI:** 10.1021/acs.jpcb.3c01085

**Published:** 2023-11-21

**Authors:** Stefan Hervø-Hansen, Jakub Polák, Markéta Tomandlová, Joachim Dzubiella, Jan Heyda, Mikael Lund

**Affiliations:** † Division of Computational Chemistry, Department of Chemistry, Lund University, Lund SE 221 00, Sweden; ‡ Division of Chemical Engineering, Graduate School of Engineering Science, Osaka University, Toyonaka, Osaka 560-8531, Japan; § Department of Physical Chemistry, University of Chemistry and Technology, Technická 5, Praha 6, Prague CZ-16628, Czech Republic; ∥ Physikalisches Institut, Albert-Ludwigs Universität Freiburg, Hermann-Herder-Straße 3, Freiburg Im Breisgau D-79104, Germany; ⊥ Lund Institute of Advance Neutron and X-ray Science (LINXS), Lund SE 223 70, Sweden

## Abstract

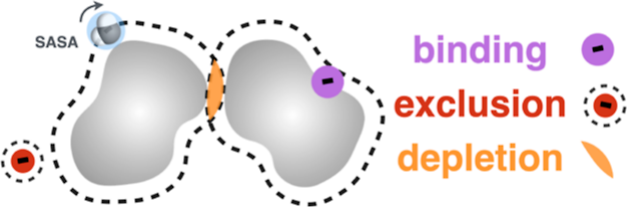

Salts affect the solvation thermodynamics of molecules of all sizes; the Hofmeister series is a prime example in which different ions lead to *salting-in* or *salting-out* of aqueous proteins. Early work of Tanford led to the discovery that the solvation of molecular surface motifs is proportional to the solvent accessible surface area (SASA), and later studies have shown that the proportionality constant varies with the salt *concentration* and *type*. Using multiscale computer simulations combined with vapor-pressure osmometry on caffeine-salt solutions, we reveal that this SASA description captures a rich set of molecular driving forces in tertiary solutions at changing *solute* and *osmolyte* concentrations. Central to the theoretical work is a new potential energy function that depends on the instantaneous surface area, salt type, and concentration. Used in, e.g., Monte Carlo simulations, this allows for a highly efficient exploration of many-body interactions and the resulting thermodynamics at elevated solute and salt concentrations.

## Introduction

In biology and chemistry, *ion specificity* is a key factor for the selective perturbation of molecular matter. The Hofmeister series^1−3^ has been a governing paradigm to explain the capacity of salts to affect protein solubility. However, Hofmeister’s discovery has a much more universal scope and applies to protein structural stability and numerous equilibria in molecular soft matter such as polymer phase transition and solubility of small molecules.^3−5^ While the ranking of salts according to their solubilizing (*salting-in*) or precipitating (*salting-out*) effect is well established, the molecular mechanisms are still under much investigation.^6−11^ The predominant hypothesis is related to preferential binding of ions to the solute surface.^6^ However, free-energy calculations^12^ suggest that water contributes to ion-specific solvation in ways that cannot be attributed to solute-ion accumulation and/or exclusion.

Furthermore, with the discovery of the reversed Hofmeister series,^4,13−15^ a mechanistic understanding of ion specificity has become even more complex. Studies of model interfaces show that ion-specific effects are related to surface net charge, polarity, and charge density of the ion.^16,17^ It is therefore increasingly important to establish predictive models that accurately capture salt-specific effects in complex (bio)molecular solutions.^11,18,19^

**Figure 1:**
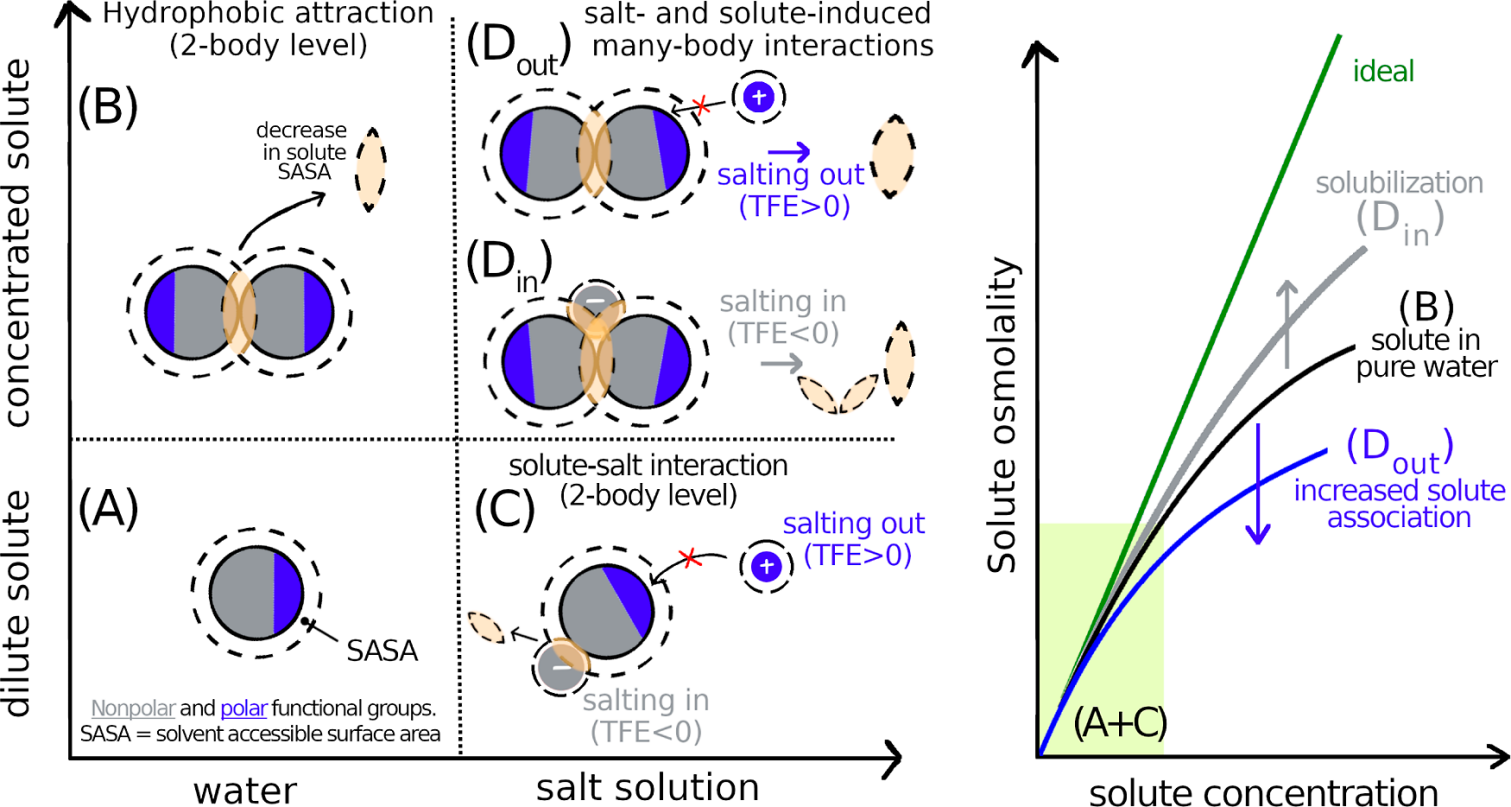
SASA-based description of regimes of a complex solute in neat water (A,B) and in aqueous salt solutions (C,D). For a fully hydrated solute at high dilution, (A) dominantly hydrophobic (gray) solute possesses a fraction of polar (blue) functional groups. SASA is depicted within the dashed line. At elevated solute concentrations of (B), hydrophobic attraction results in a decrease of SASA (pale shaded area). Increased solute association drives an osmolality decrease (right, black curve) compared to ideal behavior (right, green curve). Adding salt to a dilute solute (C), leads to (1) salting-out (TFE > 0) due to a net-ion depletion from the solute vicinity (shown by a small and strongly hydrated cation); and/or (2) salting-in (TFE < 0), due to an excess of ions in the solute vicinity (shown by a large, weakly hydrated anion binding to a hydrophobic region). At higher concentrations of (*D*), salt further affects solute–solute association. In the salting-out regime (*D*_out_), the solute is more tightly packed due to the excluded volume of depleted salt. Compared to neat water, SASA is further decreased, resulting in decrease of solute osmolality (right, blue curve). In the salting-in regime (*D*_in_), solute association allows many-body interactions with the anion, being destabilized, i.e., net solubilized. Consequently, the solute osmolality increases (right gray curve). In this case, decrease in SASA is similar to that in neat water (B), but the dissolved state is favorable due to a larger SASA region available for free-energy beneficial interactions with the anion.

The *partitioning concept*(20−24) has been particularly important for predicting how salts and osmolytes affect small and large solutes. The main idea is that the solute interacts with the surrounding environment via an inhomogeneous *solvent accessible surface area* (SASA). Upon adding the cosolute, the solvation free energy is perturbed and assumed linearly dependent on:

1. SASA
2. the concentration of the cosolute
3. a transfer-free energy (TFE), specific for the exposed area and cosolute (see Figure 1A,C)

When the cosolute is a *salt*, it has been shown that cations and anions partition independently to, e.g., air–water or hydrocarbon–water interfaces. Excluded ions exhibit a similar rank order and extent of partitioning at these nonpolar macroscopic and microscopic surfaces.^25^ The thermodynamic effect of the individual salt ions is additive to a very good approximation.^25,26^

Chemical denaturation of proteins can, e.g., be assigned to the difference in SASA between the smaller folded state and the larger denatured state. Moreover, a systematic study of proteins of various sizes confirmed that the magnitude of the chemical denaturation (e.g., by urea or guanidinium chloride) correlates with protein size.^27^ This SASA difference also drives the salt-specific effect on the solute self-association and, consequently, on the precipitation and solubilization processes. The TFE reflects the net cosolute interaction with SASA and have opposite signs for denaturating (*salting-in*) and stabilizing (*salting-out*) cosolutes. Figure 1 shows an overview of these mechanisms.

Table 1 shows several models with differing number of TFE values used to describe the cosolute effect on a chemically complex solute molecule. For example, Tanford’s original work^28^ focused on proteins and has been expanded to provide a quantitative description by assigning individual TFE values to the backbone and 20 amino acid side chains.^29−31^ Using experimental osmometry and solubility data for a large set of molecules, it has be shown that only a few functional groups (hydrophobic, aromatic, and polar) is sufficient to obtain excellent agreement with stability, equilibria, and kinetics measurements on biomolecules.^20−24,32^

**Table 1:** Various Partitioning Models and Their TFE Parameterizations^a^

reference	number of TFEs	parameterization
Tanford^28^	21	amino acid solubilities
Bolen^29−31^	21	amino acid solubilities
Record^20,21^	1–4	VPO; solubilities
this work	1–4	VPO; MD

a In the present work, we used vapor-pressure osmometry (VPO) and molecular dynamics (MD).

The originally empirical TFE-based models can be rationalized in terms of *preferential binding*(33−35) which has a solid thermodynamic foundation through Kirkwood-Buff (KB) theory. Recently, the underlying assumptions of additivity and transferability have been probed and verified in silico by molecular dynamics (MD) simulations.^36−39^ This evidence allowed a rigorous thermodynamic interpretation of salt-specific effects from experimental data as well as direct connection to MD simulation data.^9,11,40^ Recently, SASA-based arguments were critical in the development of a microscopic origin of *coil-to-globule* transition in complex mixed solvents, such as under cononsolvency conditions or in the presence of cosolvent surfactants.^41,42^

Motivated by the above partitioning models,^20,28,31^ we here devise and incorporate a SASA Hamiltonian into coarse grained (CG) Metropolis-Hastings Monte Carlo (MC) simulations, aiming to accurately capture how solute–solute interactions are affected by salts and osmolytes. The SASA-dependent potential relies on a TFE equivalent to the interaction potential parameter within the solute partitioning model. Ultimately this determines the exclusion or binding of salt species with specific functional groups, thereby affecting thermodynamic properties that are fully accessible in the new simulation scheme.

To test the model, we use caffeine, whose solubility and self-association are strongly influenced by the addition of salts.^43−48^ For example, weakly hydrated anions (ClO_4_^–^, SCN^–^, and I^–^) salted caffeine *in* the aqueous phase, while strongly hydrated anions (CO_3_^2–^, and SO_4_^2–^) salted caffeine *out*. Furthermore, caffeine self-association is promoted by strongly hydrated anions and suppressed by weakly hydrated anions.^46,49^

Caffeine has high biological significance due to it being one of the key components of coffee, tea, energy drinks, etc., making it probably the most consumed psychoactive drug worldwide. Caffeine is also one of the few naturally occurring chemicals, that has been found to have a plausible positive relationship between intake and reduced risk of Parkinson’s and Alzheimer’s disease.^50−52^ For these reasons, the chemical and physical properties of caffeine have been studied using a large variety of experimental and computational methods. The existence of oligomers limits the thermodynamic insight, obtainable from experiments performed at caffeine saturation, i.e., from solubility studies.^46,53^

In this work, we broaden the experimental window and study caffeine–salt interactions from a high dilution to intermediate concentration. To that end, we apply vapor-pressure osmometry (VPO) to aqueous solutions of caffeine at various concentrations in the presence of *salting-in* (NaSCN) and *salting-out* (Na_2_SO_4_ and NaCl) salts.

Using a new simulation model, we provide insights into the thermodynamics of *salting-in* and *salting-out* of caffeine. The model is supported by atomistic MD simulations and VPO data, which allows not only parametrization of the CG model but also gives new insights into salt-specific caffeine-salt and caffeine–caffeine interactions.

Finally, we show that under modest assumptions and known literature data on binary solutions, the results of the CG model can be fine grained to fully recover the thermodynamics of the ternary solution.

## Experimental Methodology

### Chemicals

Water used as the solvent (labeled 1 from now on) was double-distilled and treated by a Milli-Q ultrapure water purification system from Millipore. The solute (labeled 2) caffeine (Sigma-Aldrich, ≥99.0%, analysis by supplier gives purity 99.6%) was used as received without further purification. The salts (labeled 3) used were sodium chloride (NaCl, Penta p.a.), sodium sulfate (Na_2_SO_4_, Penta, >99.0%), and sodium thiocyanate (NaSCN, Fluka, ≥98.0%). NaCl was dried at 398 K for at least 12 h and stored in a desiccator.

### Vapor-Pressure Osmometry

All experiments were performed at 310 K and atmospheric pressure using a vapor pressure osmometer, an Osmomat 070 (Gonotec, Germany). VPO measures solution osmolality, which is directly related to water activity via
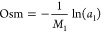
1The osmometer was calibrated before each measurement with double-distilled water and standard NaCl(aq) solution of known osmolality. The final osmolality was determined as an average of 10 readings. This protocol was found operational in our earlier study.^54^ Due to strong influence of the caffeine concentration on the caffeine–salt interaction, the experiments were designed so that the caffeine concentration was kept fixed ( = 10, 25, 50, and 75 mmol/kg), and the salt concentration was varied. Throughout this work, solely analytical or total concentrations are used, meaning that we do not distinguish between different states (monomer, dimer, etc.) of the solutes. Before each VPO experiment, ternary solutions were heated to 318 K for 1 h under constant stirring to guarantee truly homogeneous solution.

The salt–caffeine interaction was calculated according to Record et al.^20,22^ using residual osmolality  defined as
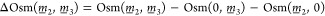
2where  is osmolality of ternary solution at  and , and  and  are osmolalities of respective binary solutions. At low caffeine and salt concentrations, the chemical potential derivative of caffeine, , is proportional to the residual osmolality^20,22^ and was described as
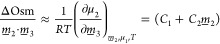
3to be consistent with eqs 1 and 13–15. In eq 3, *R* is the universal gas constant, *T* is the temperature, and *C*_1_ and *C*_2_ are constants of the linear fit to the chemical potential derivative. In case of weakly associating solutes (*C*_2_ ≃ 0), eq 3 reduces to the established relation between the residual osmolality and the salting-out constant .^20,22^ In that respect, eq 3 accounts for the effect of solute concentration on salt–solute interaction, or alternatively, *k*_s_ becomes a linear function of the solute concentration.

Finally, we remark that alternative approaches to the analysis of osmolality data are possible (raw data provided in Table S2 in the Supporting Information). This includes the generalized isodesmic model, which allows determination of the salt-specific effect on the monomer to multimer states of the solute^46^ or the application of mathematically exact KB theory of molecular association and aggregation.^55^

## Computational Methodology

### MD Simulations

All-atom MD simulations of single caffeine molecule in water and in 1 M salt solutions were performed under ambient conditions (300 K and 0.1 MPa) using Gromacs v4.5.3.^56^ Temperature and pressure were controlled by a weak velocity rescaling for the canonical sampling coupling scheme^57^ and the Parinello–Rahmann barostat,^58^ respectively. Particle mesh Ewald summation was used to account for the long-range electrostatics^59^ in combination with standard cutoff for Lennard-Jones and short-range electrostatic interactions (1 nm). All bonds containing hydrogen atoms were constrained by the LINCS algorithm,^60^ while the SETTLE algorithm^61^ was employed for water molecules.

We used the Amber 11 simulation package^62,63^ to perform polarizable all-atom MD simulation of caffeine in 1 M Na_2_SO_4_ solution^64^ (with POL3 water^65^) due to the known issues of the nonpolarizable sulfate force field.^64^ The simulation parameters were similar to those in Gromacs simulations. The major differences are in the application of Berendsen thermostat and barostat^66^ and SHAKE algorithm for dealing with constrains.^67^ Simulation time, time-step, and cut-offs were identical to those in Gromacs.

The caffeine molecule was described by the GROMOS model,^68,69^ which reproduces well the caffeine solubility in common water models. Various Hofmeister salts were employed, in particular the effect of Na_2_SO_4_, NaF, NaCl, NaBr, NaI, and NaSCN aqueous salt solutions on dissolved caffeine molecules were studied, employing the SPC/E water model.^70^ Salts were described by the force fields which were recently successfully employed in modeling of ion-specific effects in electrophoretic mobility.^71^

The leapfrog integrator with 2 fs time step was employed, and configurations were gathered every 1 ps for statistical evaluation. Systems were first minimized (to remove potential atomic overlaps) and equilibrated in terms of density and temperature during 1 ns simulation. Subsequently, the ion-distribution around caffeine was equilibrated for 20 ns, followed by 80 ns long production runs when the statistical ensemble was generated. To avoid finite size effects in the solution structure, we investigated sufficiently large systems with caffeine molecules immersed in 2760 water molecules and 55 ion pairs (1 M salt). 3D periodic boundary conditions with an equilibrium box length of 4.5 nm were used.

MD simulation data were analyzed by spatial and proximal distribution functions of ions and water in the proximity of caffeine. Caffeine-hydration and salt–caffeine interaction were quantified by KB integrals *G*_*ij*_ calculated from radial distribution functions (RDFs) *g*_*ij*_(*r*), defined in eq 4
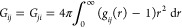
4
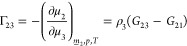
5where we are reminding that labels 1, 2, and 3 stay for solvent, solute, and salt (cosolvent), respectively. The net salt–caffeine interaction was quantified by a preferential binding coefficient, Γ_23_ defined in eq 5, where ρ_3_ is the number density of salt ions. We assigned the thermodynamic value of KB integrals to the plateau value of running KB integral *G*_*ij*_(*R*) at *R* = 1.6–2.0 nm (see the shadowed region in Figure S2).

However, in the case of nonspherical molecules, an alternative evaluation of Γ_23_ via eq 6 is preferred, which requires only the knowledge of running coordination numbers of salt ions (*N*_23_) and water (*N*_21_) to caffeine.
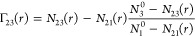
6*N*_1_^0^ and *N*_3_^0^ are the total numbers of water molecules and salt ions in the system, and the ratio  reflects the equilibrium bulk salt ion concentration to the local environment of thickness *r*. This description allows not only to quantify salt interaction with the whole caffeine molecule but also to evaluate salt-interaction contributions of individual functional groups (see Supporting Information and Figure S3).

Reminding the thermodynamic definition in eq 3, an explicit relation between Γ_23_, *k*_S_, and ΔOsm is formulated.^20^Equation 7 accounts also for the nonideality of the salt solution .
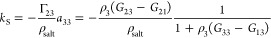
7We note that ρ_salt_ is the concentration of salt, which should not be confused with the concentration of ions ρ_3_.

### SASA Pair Potential

Dividing a molecule into *N* fragments or motifs, we assume that the individual free energy contributions are additive^72^ and proportional to their SASAs, 
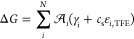
8Here γ_*i*_ is a microscopic surface tension that includes the combined effect of solute–solvent and solute–solute interactions in neat water, i.e., free of cosolutes. The term effectively describes short-range attraction stemming from, e.g., hydrophobic interactions. The second term captures the surface tension *change* as cosolutes are introduced. ε_*i*,TFE_ is a *cosolute specific* partial TFE and *c*_s_ is the molar concentration of added cosolute.

In numerical simulations, eq 8 must be evaluated for every microstate, and for large systems containing hundreds of molecules, the many-body SASA calculation becomes prohibitively expensive. We therefore adapt the approximate SASA *pair potential*: The total surface area of two spheres, *i* and *j* of radii *R* and *r* ≤ *R* and with a center-to-center separation, *d* is
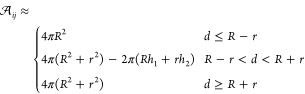
9where *h*_1_ = (*r* – *R* + *d*)(*r* + *R* – *d*)/2*d* and *h*_2_ = (*R* – *r* + *d*)(*R* + *r* – *d*)/2*d* are the heights of the two spherical caps comprising the lens formed by the overlap. In accordance with eq 8, the pair energy is calculated as  where the radii, *R* and *r*, are the particle radii plus a probe radius, while γ_*ij*_ and ε_*ij*,TFE_ are the arithmetic means of the individual values for *i* and *j*. Using this approach, γ_*ij*_ is treated as a heuristic parameter to *effectively* capture short-range attraction from, e.g., van der Waals and hydrophobic interactions.

### Monte Carlo SASA Simulation (MC-SASA)

Metropolis-Hastings MC simulations,^73^ using Faunus version 2.5,^74,75^ were conducted on CG caffeine according to the scheme presented in Figure 4, with the potential energy function
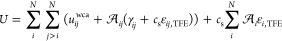
10

**Figure 2:**
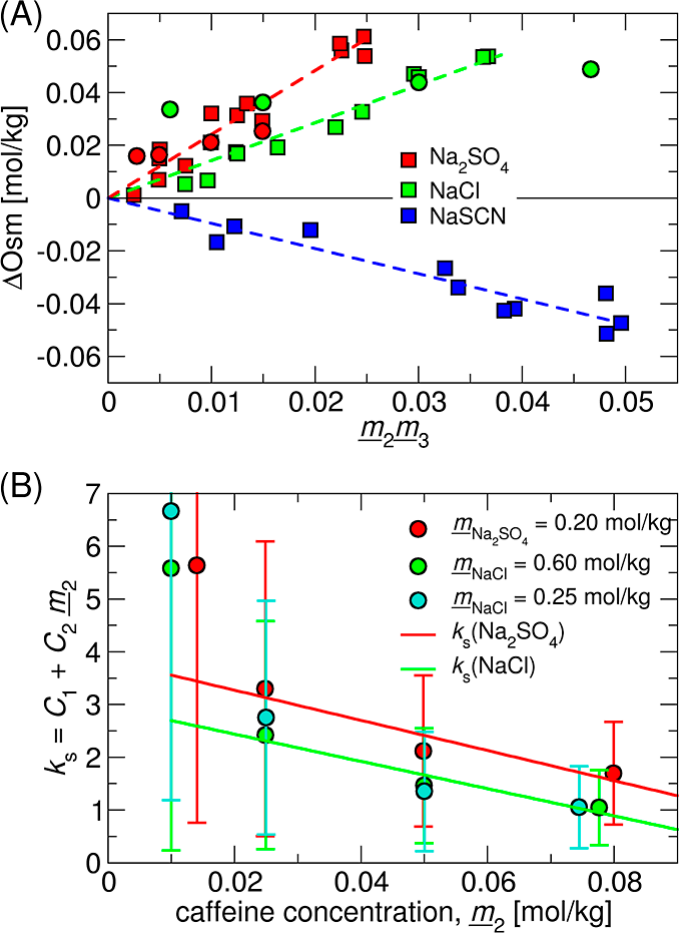
(A) Excess osmolality, ΔOsm, from VPO measurements of caffeine-salt solutions: Na_2_SO_4_ (red), NaCl (green), and NaSCN (blue). Data gathered at constant caffeine concentration  = 50 mmol/kg and varying salt concentration are presented in squares. Measurements at constant salt concentration, NaCl (0.6 mol/kg) and Na_2_SO_4_ (0.2 mol/kg) and varying caffeine concentration are shown with circles. (B) Salting-out constant, *k*_*s*_, determined at the fixed salt concentration and varying caffeine concentration . The linear fit of the *k*_s_ dependence on the caffeine concentration cf. Equation 3 is shown in red (Na_2_SO_4_) and green (NaCl) lines. Error bars are determined via error propagation at the 95% confidence level (i.e., 2 σ). The lowest caffeine concentration was omitted from the fitting. The potential role of the salt concentration of *k*_s_ is analyzed in Figure S12 in Supporting Information.

**Figure 3:**
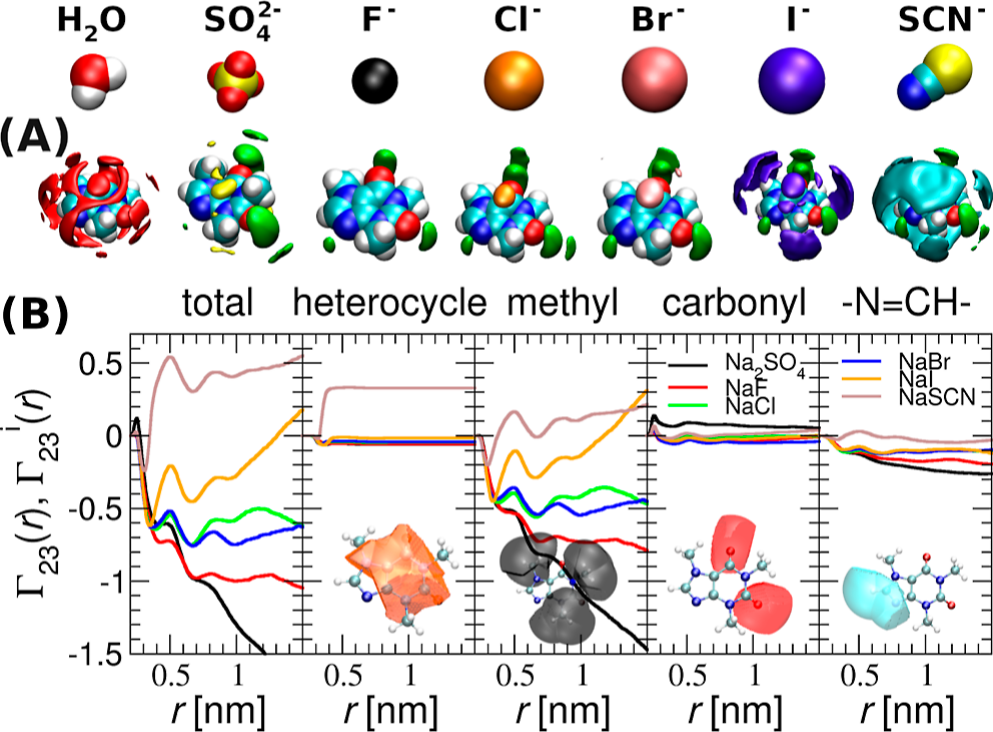
(A) Spatial distribution function (SDF) of water (leftmost panel) and ions around caffeine in salt solutions. Caffeine is depicted in a space filling representation; the distribution of water in red, sodium in green, and anions in indicated colors. The isosurfaces represent twice the bulk density for ions and 1.5 times the bulk density for water. (B) Partial preferential binding coefficients, Γ_23_^*i*^, used to quantify salt affinities to functional groups. The total preferential binding coefficients, Γ_23_ = ∑_*i*=1_^4^ Γ_23_^*i*^, are shown in the first column; the following columns show contributions from the hydrophobic heterocycle; methyl groups; carbonyl oxygen; and the —N=CH— moiety. *r* represents the proximal (closest) distance of salt to heavy atoms of a given functional group of caffeine molecule.

**Figure 4:**
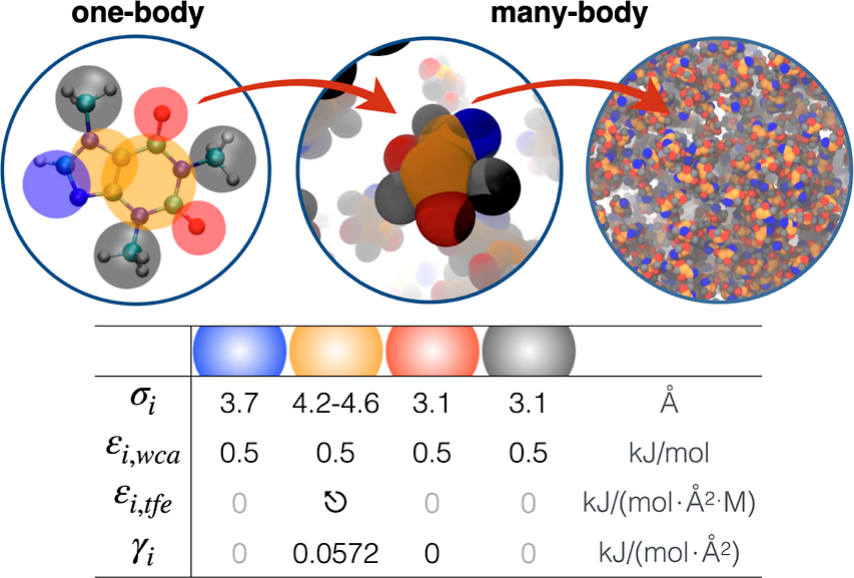
Top: Functional motifs of the atomic structure of caffeine (left) are used to generate a CG representation (middle) for investigating many-body interactions in concentrated caffeine solutions (right). In the CG models, solvent and cosolutes are implicitly accounted for, and beads correspond to the hydrophobic heterocycle (orange); methyl groups (gray); carbonyl oxygen (red); and the —N=CH— moiety (blue). Bottom: Model parameters (cf. eq 10) obtained by fitting the GCMC model to caffeine solubility data in neat water, see Supporting Information. Added osmolytes are subsequently transfer free energy, ε_*i*,TFE_.

In eq 10, the first term is the Weeks–Chandler–Anderson (WCA) potential,^76^ a shifted and truncated Lennard-Jones potential, while the second term is the approximate SASA pair potential based on TFEs, ε_*ij*_, and surface tensions, γ_*ij*_. This term is shifted to zero at nonoverlapping separations. While the first two terms are two-body pair potentials, the third and final term is a one-body potential to include the excess chemical potential of transferring a single caffeine molecule from pure water into a salt solution. This term is important only for grand canonical simulations.

The model was calibrated against experimental caffeine solubility data in neat water (*c*_s_ = 0) using 200 caffeine molecules in a cubic box, with a volume adjusted for the concentration range 0.0096–0.1116 M caffeine. The procedure is detailed in the Supporting Information, and the final parameters are shown in Figure 4.

The MC move set consisted of molecular rotation and translation with a maximum translational displacement parameter of 10 Å and a maximum rotational displacement of 1 rad. The pressure was calculated though a virtual volume move^77^ with a volume perturbation of 5 Å^3^. The osmotic coefficient, φ, was calculated from , where *p*^id^ is the ideal pressure obtained from the predetermined number density (*p*^id^ = *NRT*/*V*) and *p*^ex^ is the excess pressure obtained from virtual volume perturbations.

The effects of salt in the modulation of caffeine–caffeine interactions were investigated partly in the canonical and grand canonical ensemble. For simulations in the canonical ensemble, caffeine was simulated at different concentrations and values of the product *c*_s_ε_TFE_. The initial configuration was generated by placing caffeine molecules in a cubic box with a length of 250 Å until the desired concentration was reached. The system energy was equilibrated for 5 × 10^4^ MC iterations, followed by a sampling of the statistical ensemble for a total of 10^6^ MC iterations. The collective variables from the statistical ensemble included the caffeine–caffeine RDF, which was sampled every 10 steps with a resolution of 0.25 Å every 10 MC iterations. The excess pressure is sampled *via* a virtual volume perturbation^77^ using a isotropic scaling of the box and center of mass of caffeine, i.e., keeping the conformation of caffeine fixed, with the volume displacement being 50 Å^3^. Finally, the excess chemical potential is sampled using the Widom method^78^ with 25 inserts every 10 MC iterations. The MC move set consisted of molecular rotation and translation with a maximum translational displacement parameter of 2.5 Å and maximum rotational displacement parameter of 0.5 rad and a cluster move with molecular rotation and translation with a maximum translational displacement parameter of 7.5 Å and maximum rotational displacement parameter of 1.0 rad and threshold of 6.5 Å. For simulations in the grand canonical ensemble, caffeine was simulated at different activities and values of the product *c*_s_ε_TFE_. The initial configuration was generated by placing 2.5 × 10^3^ caffeine molecules in a cubic box with a length of 150 Å and equilibrating the system energy and particle density for a maximum of 10^7^ MC iterations. The equilibration was followed by a production run consisting of 5 × 10^7^ MC iterations, in which the statistical ensemble was generated with the sampling of the density every 1000 steps. The MC move set consisted of molecular rotation and translation with a maximum translational displacement parameter of 10 Å and maximum rotational displacement parameter of 1 rad in addition to the insertion and removal of caffeine molecules utilizing the generalized reactive MC move algorithm.^79^ The difference in excess chemical potential in *kT* units, Δμ^ex^, going from a water to an electrolyte solution, was calculated from Δμ^ex^ = ln(ρ^wat^/ρ^salt^) where ρ^wat^ and ρ^salt^ are the density of caffeine in water and salt solution, respectively, at constant caffeine activity.

### Kirkwood–Buff Analysis of Experimental and/or Simulation Data

The KB analysis was carried out by solving the set of equations eqs 11, 12 for *i* = 1 and *k* ≠ 1. These equations relate KB integrals to the chemical potential derivatives and volumetric properties of the solution^80^
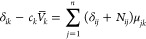
11
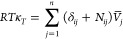
12where  is the activity derivative, δ_*ik*_ is the Kronecker δ, *n* is the number of components, κ_*T*_ is the isothermal compressibility,  are the partial molar volumes, and *N*_*ij*_ = *G*_*ij*_ρ_*j*_ are the excess coordination numbers related to KB integrals via particle number density ρ_*j*_. To obtain necessary activity derivatives, data from simulations (or experiment) were used to calculate activity coefficient of caffeine γ_2_ via fitting ln γ_2_ to
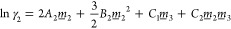
13where *A*_2_ and *B*_2_ are caffeine binary parameters, *C*_1_ and *C*_2_ are salt–caffeine interaction parameters. *A*_2_ and *B*_2_ were determined from data at  (i.e., independent of the TFE value), while *C*_1_ and *C*_2_ were specific for each TFE. Using the Gibbs–Duhem equation, approximate eq 14 for ln *a*_1_ consistently determines the form of ln γ_2_ (eq 13) and of ln γ_3_^±^ (eq 15). With that the activity derivatives of the remaining components were evaluated.
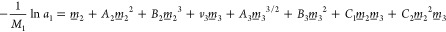
14
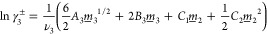
15where *A*_3_ and *B*_3_ are salt binary parameters and ν_3_ is the number of ions in the salt. Partial molar volumes and isothermal compressibility were considered independent of salt concentrations and set to values at infinite dilution.

## Results and Discussion

### VPO Investigation of Salting Out Constant of Caffeine

Salt-specific effects on sparingly to poorly soluble, small solutes are traditionally investigated by solubility measurements. Using the Setschenow equation,^43^ the impact of salt on solubility, and thus on the activity coefficient of the dissolved solute in the saturated solution, is directly probed. As the concentration of the dissolved solute at saturation is typically low, it is conveniently measured by UV–vis spectrophotometry. Here, we use VPO on homogeneous solutions to probe the salt–solute interactions as a function of the solute concentration. Optimal VPO experimental conditions are in the range from 0.1 to 1 mol/kg solute and salt concentration, which provide a good signal-to-noise ratio.

Following the protocol of Record et al.,^20,22^ we have measured osmolality of binary solutions for caffeine–water and salt–water, which were subtracted from the osmolality of the ternary solution caffeine–water–salt, according to eq 2. Solutions of various caffeine and salt concentrations were measured (see Tables S2 and S3), and the calculated ΔOsm is plotted in Figure 2A. Salt specificity leads to different slopes (see dashed lines), which fit well with the scattered experimental data obtained at fixed caffeine concentration (squares,  = 50 mmol/kg) via eq 3, and relate to the salting-out constants, summarized in Table 2. The VPO data follow the traditional Hofmeister series^9^ with Na_2_SO_4_ being the most potent salting-out salt, followed by NaCl. Salting-in behavior is observed for NaSCN.

**Table 2:** Salting Out Constants and Their Caffeine Concentration Dependence (if Available) as Determined from VPO Data in Figure 2A,B, Respectively^a^

	Na_2_SO_4_	NaCl	NaSCN
*C*_1_	3.84	2.95	*n.a*
*C*_2_	–28.59	–25.83	*n.a*
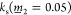	2.41	1.43	–0.96

a *C*_*i*_ parameters are in units of reciprocal molality concentration of appropriate power, and  is in mol/kg.

Taking a closer inspection, outlying points (circles) appear for Na_2_SO_4_ and NaCl (not measured for NaSCN). These represent measurements at significantly different caffeine concentrations (10–80 mmol/kg). This can be accounted for by fitting *via*eq 3. The results are plotted in Figure 2B where we note that uncertainties increase with the decreasing caffeine concentration due to increasing noise and error propagation. For NaSCN, large uncertainties at low caffeine concentrations prohibited the fitting of *via*eq 3.

Salts have the strongest effect at infinite caffeine dilution and weaken approximately linearly with the increasing caffeine concentration. The parameters *C*_1_ and *C*_2_ of the presumed linear effect in the caffeine concentration are summarized in Table 2. Comparison of salting-out constants at two NaCl concentrations indicates that this effect is due to the variation in the caffeine concentration. The effect of the salt concentration on *k*_s_ is analyzed in detail in Figure S12 in the Supporting Information and is found to be significantly smaller than that of the caffeine concentration.

Observed weakening of the salting-out effect (*C*_1_ > 0) with an increasing caffeine concentration (*C*_2_ < 0) may be also interpreted as a strengthening of caffeine–caffeine self-association in the presence of salting-out salts and is consistent with earlier findings based on caffeine solubility and caffeine partitioning study.^43^

In other words, our data show that the strength of the salting-out effect of the salt in general depends on the solute state (presumably due to differences in SASA, see the next sections). This includes, e.g., native vs denatured state of proteins; coil vs globular state of polymers; or solution structure of solute molecules in the aqueous environment. For caffeine, the latter is manifested as a concentration-dependent self-association.

The correlation between solubility and self-association was reported for hydrophobic molecules (e.g., methane and neopentane) in earlier computational study.^81^ Moreover, within a statistical-thermodynamic approach, a rigorous relation between the salting-out constant and salt-effect on self-association (virial coefficient) was derived, at least for small hydrophobic solutes.^82^ In the current work, we use novel MC-SASA simulations to shed light on the experimental data and explore the relation between the salting-out effect and self-association for complex caffeine molecule.

Salting-out constants determined from the slopes in Figure 2A are significantly higher, compared to previously published data.^43,46^ The difference is, however, only *apparent* and can be rationalized with the following arguments, exemplified for NaCl: the salting-out constant decreases with the increasing caffeine concentration, thus reaching its lower limit at caffeine saturation. Caffeine solubility steeply increases with temperature, i.e., it is approximately two times higher at 37 °C than that at 25 °C.^83^ The VPO experiments are thus performed at a rather far from saturation. Assuming that the Setschenow constant is invariant in this temperature window, the estimated caffeine solubility is approximately 0.16 mol/kg at 37 °C in 0.6 mol/kg NaCl.^43,83^ Extrapolating the results in Figure 2B to saturation, the salting-out constant in NaCl is approximately zero, which recovers the literature value.^43,46^

### All-Atom MD Reveals Anion-Specific Binding Sites of Caffeine

To gain an understanding of the origin of the affinity of various ions to the caffeine molecule, we have conducted all-atom MD simulations of caffeine in aqueous solutions of sodium salts. The anions ranged from salting-in thiocyanate (NaSCN) over weakly salting-out chloride (NaCl) to strongly salting-out sulfate (Na_2_SO_4_).

First, detailed insights into the very vicinity of the caffeine molecule are obtained by employing the spatial distribution function (SDF), as shown in Figure 3A. The spatial clouds represent the sites of significantly enhanced probability of water oxygen, anions, and sodium in the proximity of caffeine molecule.

In agreement with the Hofmeister series for anions, weakly hydrated anions (SCN^–^, I^–^) are enriched at hydrophobic sites. The latter constitute the three methyl groups and the two heterocyclic aromatic rings. In contrast, the more strongly hydrated SO_4_^2–^ and F^–^ occur in the caffeine vicinity less than water, i.e., they are depleted. Na^+^ is present near the partially negatively charged amide (C=O) and imine (—N=CH—) moieties. The magnitude of the sodium affinity in the individual salts reflects the excess of its counteranion due to electrostatic correlations. The remainder of the caffeine surface lacks specific interaction sites for salt and water. In summary, anion enrichment or depletion is driven by interactions with the hydrophobic regions of caffeine.

In agreement with previous studies,^68,69^ the SDF also shows that caffeine is well hydrated by water alone, which is somewhat counterintuitive considering the weak hydrogen-bonding capacity. Tighter hydration is primarily due to the heterocyclic face (owing to effective π–OH interactions) and the amide and imine groups, while methyl groups are poorly hydrated.

The thermodynamic measure of salt excess or depletion near caffeine is captured by the salting-out constant *k*_S_. Its origin from the microscopic solution structure stems from KB theory.^84^ To that end, we have calculated RDFs, evaluated KB integrals, and calculated the preferential binding coefficient Γ_23_ and salting-out constants. These net-salt effects are summarized in Figures S1 and S2, Table S1, and discussed in detail in the Section S1.

Following our observation in SDFs, we decomposed the caffeine surface into distinct functional groups and evaluated partial proximal distribution functions in Figure S3. From this, we calculate the net preferential binding coefficient, Γ_23_ (first column of Figure 3B) and introduce partial preferential binding coefficients Γ_23_^*i*^ of salts to individual functional groups of caffeine (second to fifth column). The magnitudes of Γ_23_^*i*^ in Figure 3B show that the preferential binding to methyl groups (third column) controls the salt specificity. Only SCN^–^ displays some affinity to the heterocyclic aromatic rings (second column). Although some salt specificity is also present in the interactions with amide and imine moieties, quantitatively, these are of minor importance. Strictly speaking Figure 3B presents running preferential binding coefficients Γ_23_(*R*), from which plateau region (≈1.5 nm from center of mass, or ≈1.0 nm from the surface of caffeine molecule) the thermodynamic value Γ_23_ is determined (see also Figure S2).

To summarize, all-atom MD simulations allow us to calculate salting-in/salting-out capability of salts in the limit of infinite dilution of caffeine, where the contributions of caffeine–caffeine interactions are absent. A logical continuation toward the modeling of salt-specific effects at finite caffeine concentrations is the application of an appropriate CG model, which is present in the following sections.

### CG Model of Caffeine Using Thermodynamic Data

To investigate the impact of the self-association equilibrium of caffeine on salting-in/salting-out capabilities of salts, a CG model has been constructed. The coarse graining has been conducted using a combined top-down and bottom-up approach. The structural results of anion–caffeine association, obtained from the all-atomic MD simulations, was used to create unified atoms, in which individual methyl groups and heterocycles, constituting the nonpolar groups of caffeine, the imine group, and carbonyl oxygen, constituting the polar groups of caffeine, can be represented by single spheres. The resulting coarse-graining scheme is visualized in Figure 4.

To reproduce the caffeine–caffeine interactions, we calibrated our CG model using experimental data using osmotic coefficients, which represented the average attraction/repulsion between caffeine molecules in salt-free water.^85,86^ The parametrization of caffeine was conducted by adjusting the atomic surface tension, γ_*i*_, at a constant value of ϵ_*i*_ = 0.5 kJ/mol for the WCA potential (see eq 10), to reproduce the experimental osmotic coefficients. From atomistic simulations,^68^ nuclear magnetic resonance spectroscopy,^87^ and small angle neutron scattering^88^ experiments, it has been established that the primary mode of association in caffeine oligomerization at room temperature is face-to-face stacking. Consequently, we choose to simplify our model further by limiting the number of attractive sites to the two CG spheres constituting the heterocyclic aromatic groups. The osmotic coefficients obtained from simulations using the best parameter for the atomic surface tension (γ = 0.0572 kJ/mol/Å^2^) to reproduce the experimental data has been visualized in Figure S4.

At first glance, the osmotic coefficients obtained from the simulation yield good agreement with the experimental data over the available caffeine concentration range. Deviations, however, between experimental data and simulated data do occur at high caffeine concentrations, suggesting that the CG Hamiltonian causes too strong attraction between caffeine molecules. This is likely due to the pair potential being much softer, being nearly linear compared to potentials usually employed for short-ranged attraction. This has the implication that the attraction persists over a longer range compared to, for example, a sixth-inverse-power of Lennard-Jones potentials (see Figure S7). This range is related (i) to the probe radius when calculating the surface area and (2) the magnitude of the WCA potential determined by ϵ. Varying the number of TFE sites to also include the methyl groups by equal and half strength compared to the heterocycles proved to yield highly equivalent results (Figure S8). While the model possessing more TFE sites reproduces the osmotic coefficient better at the increasing caffeine concentration, it will be shown next that the model utilizing only two TFE sites is sufficient to capture the important structural and thermodynamic properties.

In agreement with experimental observations, the most probable mode of caffeine–caffeine association is face–face stacking, with branching being much less probable.^88^ The stacking to branching ratio can most likely be controlled by the introduction of more interaction sites (CG atoms with nonzero tension), such as the methyl groups. However, the employed model captures the thermodynamics of the caffeine–caffeine interactions. Looking at the RDF between the caffeine molecules (Figure S5), we find the KB integral, *G*_22_, being equal to approximately 20 × 10^3^ Å^3^ in agreement with the experimental data from the literature.^85^ Consequently, our model possesses qualitative correctness with the ability to capture essential features, laying the foundation for employing the model in investigating how salt alters caffeine’s liquid structure.

### Salting-In and Salting-Out from Implicit Salt Hamiltonian

#### Comparison with Solubility Experiments

With the CG model parametrized to reproduce experimental osmotic coefficients in the 10–100 mM concentration range, we continued to parametrize our model to include salt effects. To do so, we searched for TFE values at constant activity of caffeine that can reproduce experimental differences in the excess chemical potential of caffeine in salt solution at a specific salt concentration and pure water. Within the theory of solute solubility, a convenient experimental measure, pseudo chemical potential (pcp, μ*) was introduced by Ben-Naim.^89^

**Figure 5:**
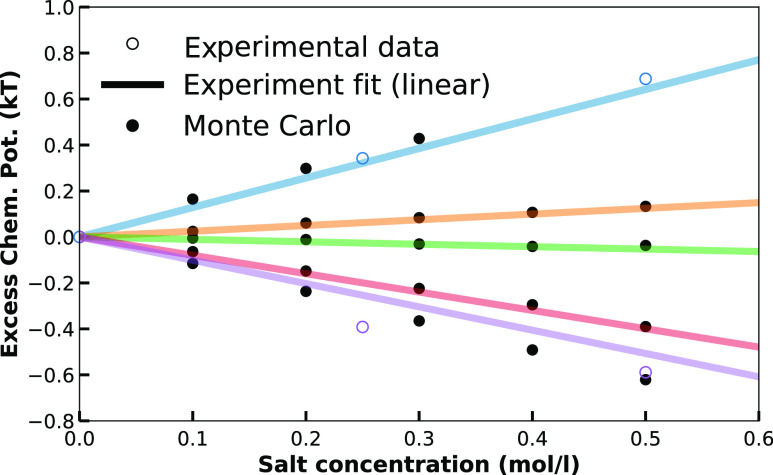
Excess chemical potential of caffeine in salt solutions compared to neat water at a constant molar activity of caffeine (26 mM). Experimental^46^ and MC-SASA can be mapped onto each other through salt-specific TFE-values (ε_TFE_ in units of kJ mol^–1^ Å^–2^ M^–1^): Na_2_SO_4_: 0.0178 (blue), NaCl: 0.003 (orange), NaBr: −0.001 (green), NaSCN: −0.008 (red), and NaClO_4_: −0.0125 (purple). Nonspecific simulation parameters are taken from pure caffeine/water solutions; see Figure S4.

The pcp values of caffeine were determined by Shimizu for different cosolutes and cosolute concentrations from caffeine solubility data.^46^ Since the experiment probes caffeine solutions near saturation, ideally, we are interested in the chemical potential of the liquid phase when the liquid and aggregated phases are in equilibrium at constant temperature and pressure. However, methodologies involving aggregated or solid states of matter in equilibrium with the liquid state are usually not preferred due to either high computational costs (free energy calculations and extended ensembles) or the transition being a rare event, yielding poor statistics (direct sampling). Consequently, TFE parametrization was carried out at a caffeine molar activity of 0.026 in the grand canonical ensemble which is equivalent to a caffeine concentration of 69 mM in pure water (ε_TFE_ = 0). This enables us to sufficiently sample converged results without the aggregated phase being sampled. The excess chemical potential, μ^ex^, obtained from simulations yielding the best agreement with the experimental data are shown in Figure 5.

From the simulation, we obtain the excess chemical potential, while the experimental data involve the pseudo chemical potentials. The two are related by μ^ex^ = μ* – *RT* ln(*q*) where *q* is the internal partition function containing all rotational degrees of freedom. However, for molecules with small conformational ensembles and assuming that the addition of cosolute does not influence that ensemble, the change in pcp can be assumed equal to that in excess chemical potential.^84^

Different unique TFE values can effectively reproduce the chemical potential of caffeine in salt solutions at different concentrations. Within our TFE model, salting-in and salting-out effects of salts are solely controlled by the sign of the TFE value. A consequence of our model is the linear dependency of excess chemical potential on the additive concentration; however, the analyzed experimental data by Shimizu is fitted to a second-order polynomial, with the fitting parameter in the linear term being identified as the Setschenow constant, while the quadratic term serves to capture nonlinear effects over a wide cosolute concentration range. Conducting linear regression analysis and hypothesis testing on the available experimental data, however, reveals it to be insufficient including the quadratic term (linear *r*^2^ > 0.96 for all salts and sucrose), and a linear fit is substantial to describe the experimental data as also suggested by the squared regression coefficients, which adds to the validity of our model.

**Figure 6:**
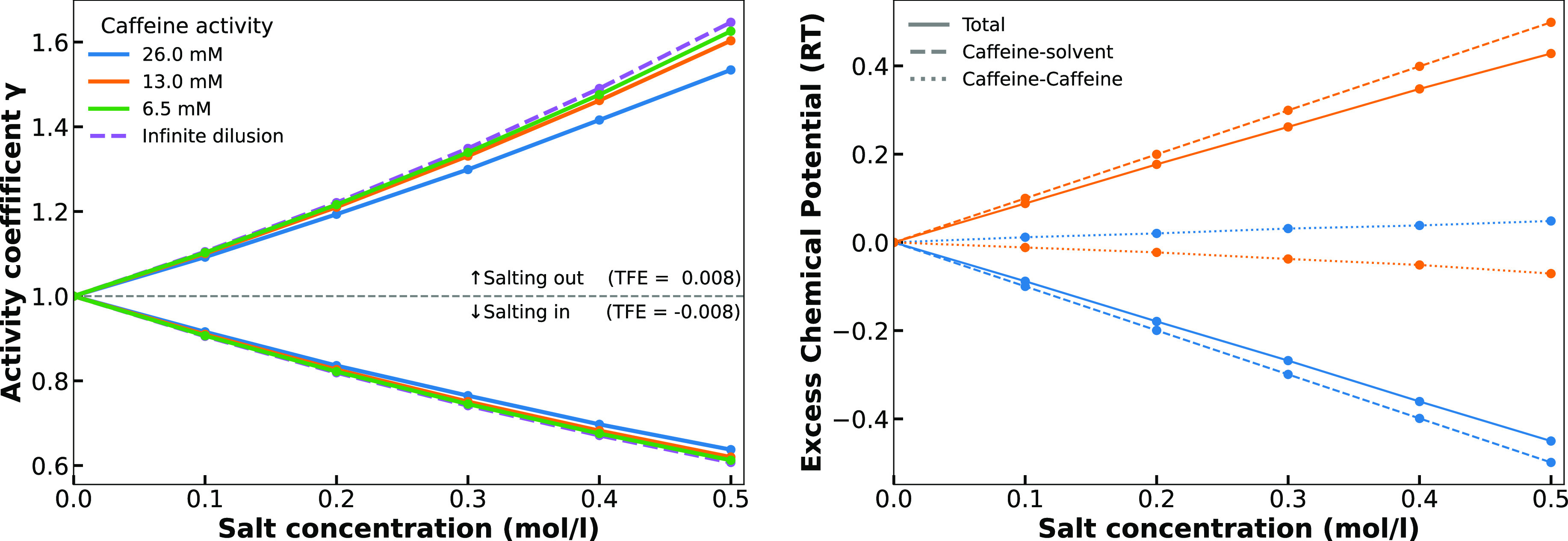
*Left*: Activity coefficient for caffeine (set to unity in salt-free solution) as a function of salt concentration (ε_TFE_ = 0.008 kJ mol^–1^ Å^–2^ M^–1^ for salting-out and ε_TFE_ = −0.008 kJ mol^–1^ Å^–2^ M^–1^ for salting-in) for three caffeine activities sampled by MC simulation and infinite dilution calculated analytically using the Hamiltonian in eq 10. *Right*: Decomposition of the caffeine-solvent and caffeine–caffeine contribution to the difference in excess chemical potential between caffeine in salt solution and caffeine in pure water. The orange curve corresponds to salting out conditions (ε_TFE_ = 0.008), while the blue curve is equivalent to salting in (ε_TFE_ = −0.008). The molar activity of caffeine is 0.026.

The excess chemical potential obtained from the grand canonical simulations possesses two contributions: (a) a one-body term representing the TFE of a caffeine monomer from pure solvent to a solvent with an additive solution and (b) a caffeine–caffeine interaction term. These two contributions are shown in Figure 6.

Within our model, the TFE of a caffeine monomer from water to water with an additive opposes the free energy contribution from caffeine–caffeine association. This observation is independent of the sign of the TFE value, while it does, however, determine whether the individual contributions are positive or negative. Conceptually, this is in agreement with the mechanistic understanding of the Hofmeister effect: ions causing salting-in are solute binding species that increase repulsion between solute molecules due to electrostatic repulsion; ions causing salting-out are solute excluding species that increase the attraction between solute molecules due to an enhanced hydrophobic effect by strongly hydrated species.

Within our model, electrostatics are *implicitly* incorporated into the TFE, assuming that long-range repulsion can be negligible due to short screening lengths at high salt concentrations. The model could however be extended to *explicitly* include electrostatics by a reactive MC move, in which CG caffeine atoms can change charge upon binding of an (implicit) anion.^90^

### Caffeine Self-Association Modulates the Effect of Salt

The CG model is now tuned to reproduce thermodynamic observables related to the average attraction and repulsion between caffeine molecules in water and the electrolyte solution. We now continue to investigate the effect of elevating the *caffeine concentration*.

Caffeine possesses a self-association equilibrium, characterized by a specific binding mode between the heterocycle faces of the caffeine molecules. Consequently, by changing the caffeine concentration (or solvent quality, see Figure S6), the relative population of caffeine monomers and caffeine oligomer states is perturbed. Figure 6 shows simulated activity coefficients of caffeine in water with the increasing cosolute (salt) concentration. The effect of cosolute diminishes with increasing activity of caffeine, particularly for positive TFE values which is trivially due to Boltzmann weighting of the TFE values in the Hamiltonian. Experimentally, this has great importance in terms of design and choice of methodology. For determining Setschenow coefficients, common methods include organic-aqueous phase solute partitioning, liquid–solid phase partitioning, and VPO. Each method, however, operates in different concentration regimes which is problematic since our findings suggest that the Setschenow coefficient in general depends on the solute concentration. Consequently, different techniques may yield different Setschenow coefficients, solely because of differences in the solute concentration.

### Recovering the Full Solution Thermodynamics

We now completely recover the thermodynamics of aqueous caffeine-salt solution using KB theory, applying only modest assumptions on implicitly treated components. To that end, we have used MC-SASA for a series of caffeine and salt concentrations,  = 0–0.05 mol/kg ×  = 0–0.5 mol/kg, and two types of salt, i.e., salting out (ε_TFE_ = 0.01 kJ mol^–1^ Å^–2^ M^–1^) and salting in (ε_TFE_ = −0.01 kJ mol^–1^ Å^–2^ M^–1^). The direct output of MC-SASA is the excess chemical potential of caffeine (μ_2_^ex^ = *RT* ln γ_2_), as summarized in Table S4. Data were globally fitted to eq 13, yielding *A*_2_, *B*_2_, *C*_1_, and *C*_2_. Known activity data for binary water-salt solution provided parameters *A*_3_ and *B*_3_, and standard state partial molar volumes  at 37 °C were employed for dissolved species and water.^91,92^ The approximation of constant  is justified as the KB integrals are only weakly dependent on  but are dominated by changes in activity data.^80^ A complete set of coefficients for the thermodynamic description of the salt-caffeine solution in two representative salts is summarized in Table 3.

**Table 3:** Parameters of the Thermodynamic Model of Caffeine–Salt Solutions^a^

	ε_TFE_ = −0.01	ε_TFE_ = 0.01	description
*A*_2_	–7.2399	–7.2399	caffeine–caffeine
*B*_2_	24.9741	24.9741	caffeine–caffeine
			
*A*_3_	–0.6216	–0.3506	salt–salt
*B*_3_	0.4754	0.2087	salt–salt
			
*C*_1_	–1.1145	0.8061	caffeine–salt
*C*_2_	6.1971	–8.0176	caffeine–salt–caffeine
			
*V̅*_1_	18.14	18.14	water partial molar volume
*V̅*_2_	145.9	145.9	caffeine partial molar volume
*V̅*_3_	40.96	16.62	salt partial molar volume

a *A*_*i*_, *B*_*i*_, and *C*_*i*_ are in units of reciprocal molality of appropriate power. Partial molar volumes,  are in in cm^3^·mol^–1^. ε_TFE_ = −0.01 kJ mol^–1^ Å^–2^ M^–1^ and ε_TFE_ = 0.01 kJ mol^–1^ Å^–2^ M^–1^ represent salting-in and salting-out salts, respectively. *A*_2_, *B*_2_, *C*_1_, and *C*_2_ are obtained by fitting the MC-SASA data (Table S4) to eq 13.

Employing the KB-inversion procedure using eqs 11 and 12, Figures S9 and S10 show all six KB integrals for the two types of salts over the range of caffeine and salt concentrations. Note that KB integrals carry information about the net-solution structure. The most insightful KB integrals are those involving caffeine, i.e., *G*_12_, *G*_22_, and *G*_23_. These are presented for the two types of salts in Figure S11 and discussed in detail in the Supporting Information.

**Figure 7:**
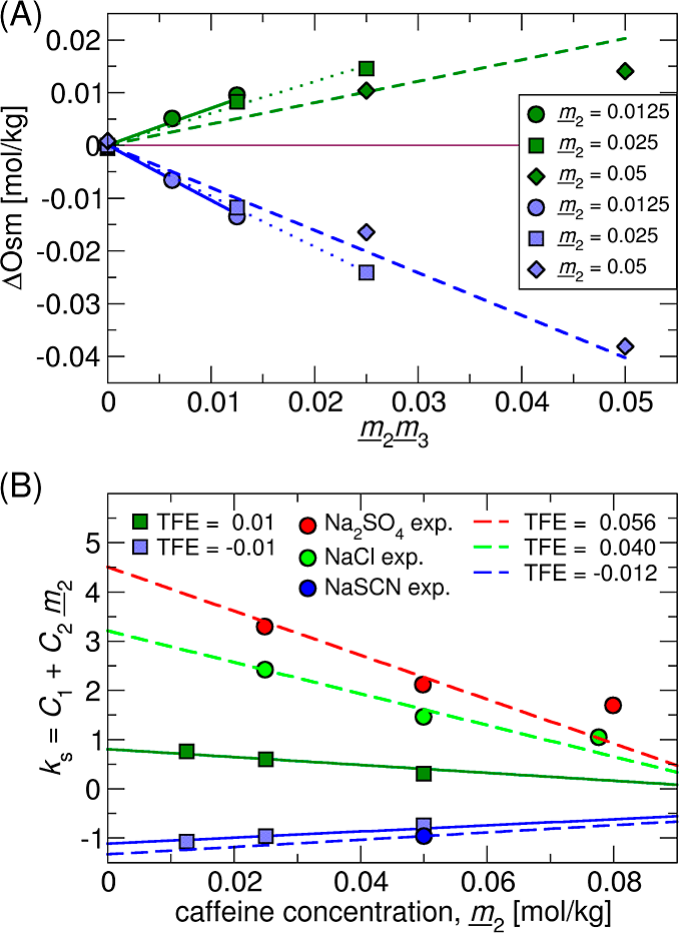
(A) ΔOsm from MC-SASA of caffeine solute using salting-out (ε_TFE_ = 0.01 kJ mol^–1^ Å^–2^ M^–1^, in green) and salting-in (ε_TFE_ = −0.01 kJ mol^–1^ Å^–2^ M^–1^, in blue). Points present raw MC-SASA data (i.e., μ_2_^ex^, Table S4), while lines represent a global fit of MC-SASA data (i.e., with parameters in Table 3). (B) Salting-out constant (cf. eq 3) as a function of the caffeine concentration for ε_TFE_ = −0.01 kJ mol^–1^ Å^–2^ M^–1^ (light blue) and ε_TFE_ = 0.01 kJ mol^–1^ Å^–2^ M^–1^ (dark green) raw MC-SASA data (squares) along with analytical curves from Table 3 (full lines). Experimental data for Na_2_SO_4_ (red circles), NaCl (green), and NaSCN (blue) from Figure 2B fitted by the *universal* MC-SASA curves (dashed lines) with a single TFE parameter determined (see the legend).

A practically important outcome of the MC-SASA simulations is the construction of an *in silico* VPO experiment, which allows evaluation the  and thus the caffeine–salt interaction. Analogous to Figure 2, the simulated Figure 7A shows raw ΔOsm data from individual MC-SASA calculations (points) along with the slopes, obtained from eq 3 for a series of caffeine concentrations. Although the two salts differ only in the TFE-sign, the two responses in ΔOsm are not mirror images.

Since only the *product* ε_TFE_*c*_s_ enters the MC-SASA calculation, the simulation results are universal and can be used to recalculate ΔOsm curves for the arbitrary TFE value. Figure 7B clearly shows the caffeine concentration dependence of the salting-out constant, *k*_S_, as evaluated from raw ΔOsm data together with the curves evaluated via eq 3. Finally, the experimental data for Na_2_SO_4_ (red cicrcle), NaCl (green circles), and NaSCN (blue circles) from Figure 2B were fitted by the *universal* MC-SASA curves (dashed lines), and the caffeine–salt interaction parameter (TFE value) was determined. The relation between *C*_1_ and *C*_2_ was recently predicted by the statistical-thermodynamic approach for small nonpolar solutes.^82^ In this work, a simple MC-SASA scheme (see results in Figure 7) captures higher order salt-solute effects, which originate in salt-specific effective interaction with different surface types (functional groups) of complex solutes.

Examplified by caffeine, the present results show that the MC-SASA approach quantitatively captures the changes of the solute chemical potential in the presence of additives. Moreover, the ability of the model to faithfully account for many-body effects, i.e., solute association, points toward its broad applicability from modeling of protein conformational changes up to protein–protein interactions in crowded environments.

## Concluding Remarks

Using VPO and molecular scale computer simulations, we have investigated how specific ion effects operate over different *solute and salt* concentration regimes. Experimental observations were rationalized by introducing an implicit-water, implicit-salt MC model, which uses structural details of solute hydration and solute–salt interactions through the solvent accessible surface area (MC-SASA). Applied to the chemically complex caffeine molecule and salts from the extreme ends of the Hofmeister series, the new method gives quantitative insights into *salting-in* and *salting-out* of caffeine over a range of solute and cosolute concentration regimes.

To fine-grain our model, we present a KB theory-based protocol in which complete thermodynamics of the ternary solution is recovered. All KB integrals at any composition are determined from MC-SASA simulation data, employing only modest assumptions on implicitly treated components.

The new MC-SASA algorithm captures the modulation of the magnitude of the *salting-in* or *salting-out* effect with the increasing solute concentration in accordance with the new VPO data. The development of our MC-SASA model was motivated by the existing partitioning concept^20−24,28^ and can be regarded as a generalization where many-body effects are taken into account. While here applied to caffeine salt solutions, the methodology can be expanded to large complex systems in which the implicit treatment of solvent and salt becomes highly efficient. This includes, but is not limited to, polymers, proteins, or protein–ligand complexes at dilute conditions, as well as to concentrated solutions of biomolecules in the presence of crowders.

## Supporting Information Available

Additional explanations, tables, and plots (PDF)

## Notes

An Electronic notebook for running simulations, analyzing, and plotting the presented data is available at Zenodo, https://doi.org/10.5281/zenodo.10122308.
