# Supplementary material for: Salt Effects on Caffeine across Concentration Regimes
Source: J Phys Chem B. 2023 Nov 21;127(48):10253–65. doi: 10.1021/acs.jpcb.3c01085 (PMC10702535; doi:10.1021/acs.jpcb.3c01085)
Supplement: Supplementary file 1 — jp3c01085_si_001.pdf [file jp3c01085_si_001.pdf]

Supporting Information: Salt Effects on Caffeine Across Concentration Regimes

Stefan Hervø-Hansen[1], Jakub Polák[2], Markéta Tomandlová[2], Joachim Dzubiella[3], Jan Heyda[2], Mikael Lund[1]

October 23, 2023

1 Supplementary materials

S1 All-atom MD: Solution structure near caffeine, preferential binding and salting-out constant

We remind that the following labeling convention is used: water(1), caffeine(2), salt(3).

Figure S1 presents the most prominent radial distribution functions (RDFs), *i.e.*, those which involve caffeine molecule. The salt distribution around caffeine ($g_{23}(r)$) should be compared with caffeine hydration ($g_{21}(r)$). The solvation-hydration competition is the origin of the preferential binding. Unlike in the experiment, we can decompose salt distribution to individual ions. Anions, present in $g_{2-}(r)$, follow the Hofmeister series, with visible enrichment of SCN^- and I^- at short distances on one end, and significant depletion of F^- and SO_4^{2-} on the other end of the series. Distributions of Na^+ cations are qualitatively similar and, due to electrostatic correlations, they quantitatively follow the order of anions.

Net caffeine-ion and caffeine-water interactions, Kirkwood-Buff integrals G_{ij} (KBIs), are obtained by integration via Eq. (S1), and are summarized in Figure S2. While the figure presents the running KBIs, the thermodynamic limit can be read from the plateau value at larger distances (highlighted by the shaded area). The salt-caffeine (G_{23}) follows the order of salts in Hofmeister series. It might be anticipated that the caffeine-water affinity G_{21} depends on the quality of salt only to a minor extent. The net preferential binding coefficient Γ_{23} is evaluated via Eq. (S2). Its salt-nonideality corrected analogue $\Gamma_{23}a_{33}$ (Eq. (S5)) is directly related to the thermodynamic Setschenow coefficient or salting-out constant also follows the Hofmeister series [1, 2]. The evaluated KBIs, Γ_{23}, a_{33} and k_S are summarized in Table S1.
Kirkwood-Buff integrals, presented in Figure S2, are calculated from radial distribution functions, via Eq. (S1), assigning its value to the plateau value of running KB-integral $G_{ij}(R)$ at R =1.6–2.0 nm (see shadowed region in Figure S2). Their knowledge can be used to calculate preferential binding coefficient Γ_{23}. Particularly simple relation, Eq. (S2) holds in case of single solute (caffeine) molecule in binary mixed solution, i.e., water-salt. In this case Γ_{23} reflects the competition of caffeine solvation (G_{23}) and hydration (G_{21}), and it is proportional to salt ion number density ρ_3.

$$G_{ij} = G_{ji} = 4\pi \int_0^\infty (g_{ij}(r) - 1)\, r^2 \mathrm{d}r \approx 4\pi \int_0^R (g_{ij}(r) - 1)\, r^2 \mathrm{d}r = G_{ij}(R) \tag{S1}$$

$$\Gamma_{23} = \rho_3 (G_{23} - G_{21}) \tag{S2}$$

On the practical side of the calculation, however, in case of non-spherical molecules it is useful to apply an alternative evaluation of the preferential binding coefficient ($\Gamma_{23}(r)$) via Eq. (S3), which requires only the knowledge of coordination numbers of salt ions (N_{23}) and water (N_{21}) to caffeine.

$$\Gamma_{23}(r) = N_{23}(r) - N_{21}(r)\frac{N_3^0 - N_{23}(r)}{N_1^0 - N_{21}(r)} \tag{S3}$$

$$\Gamma_{23} = -\left(\frac{\partial \mu_2}{\partial \mu_3}\right)_{\underline{m}_2,p,T} \tag{S4}$$

$$k_\mathrm{S} = -\frac{\Gamma_{23}}{\rho_\mathrm{salt}} a_{33} = -\frac{\rho_3(G_{23} - G_{21})}{\rho_\mathrm{salt}} \frac{1}{1 + \rho_3(G_{33} - G_{13})} \tag{S5}$$

where N_1^0 and N_3^0 is the total number of water molecules and salt ions in the system, and the ratio $\frac{N_3^0-N_{23}(r)}{N_1^0-N_{21}(r)}$ reflects the equilibrium bulk salt ion concentration to the local environment of thickness r. k_S is the salting out constant, ρ_{salt} is concentration of salt (not ions), and $a_{33} = \left(\frac{\partial \ln a_3}{\partial \ln \rho_3}\right)_{T,p}$ account for the nonideality of the salt solution. The connection to macroscopic thermodynamics, i.e., changes of caffeine chemical potential μ_2 is obtained by simple relation eq. (S4), from which follows the most prominent one, i.e., the salting-out constant k_S.

Table S1: Summary of KB-integrals G_{ij} (in nm^3), preferential binding coefficients Γ_{23} (particle per particle) and salting out constants k_S (nm^3) as determined from all-atom MD simulations for investigated salt solutions. KB-integrals were evaluated from radial distribution functions (Figure S1), respectively from shadowed plateau region of running KB-integrals (Figure S2).

System	ρ_{salt} [nm^{-3}]	G_{21}	G_{23}	Γ_{23}	k_S	G_{33}	G_{31}	a_{33}
Na_2SO_4	0.33	-0.2	-1.5	-1.28	2.72	0.4	-0.04	0.70
NaF	0.44	-0.2	-1	-0.70	0.95	0.8	0.02	0.59
NaCl	0.60	-0.2	-0.7	-0.60	0.95	0	-0.04	0.95
NaBr	0.60	-0.2	-0.8	-0.72	1.29	-0.1	-0.04	1.08
NaI	0.60	-0.2	-0.3	-0.12	0.27	-0.25	-0.04	1.34
NaSCN	0.60	-0.2	0	0.24	-0.46	-0.15	-0.04	1.15

Note on relation between single ion and salt KBIs:
Although the RDFs of individual ions differ (e.g., compare $g_{2-}(r)$ and $g_{2+}(r)$), their excess around caffeine (or water) molecule must obey electroneutrality condition (see equations below). Assuming a salt $A_{\nu_+}B_{\nu_-}$ of concentration ρ_s, which dissociate to ν_+ cations of a charge z_+ and ν_- anions of a charge z_-, generate cation concentration $\rho_+ = \nu_+\rho_s$, anion concentration $\rho_- = \nu_-\rho_s$ and total ion concentration $\rho_3 = (\nu_+ + \nu_-)\rho_s$. Consequently the KBIs are not independent, but follow simple relations [3]. Figure S2 documents that our all-atom MD results obey these conditions very well.

$$z_+N_{2+} = z_+G_{2+}\rho_+ = z_-G_{2-}\rho_- = z_-N_{2-}$$
$$N_{23} = N_{2+} + N_{2-}$$
$$G_{23} = \frac{\nu_+G_{2+} + \nu_-G_{2-}}{\nu_+ + \nu_-}$$

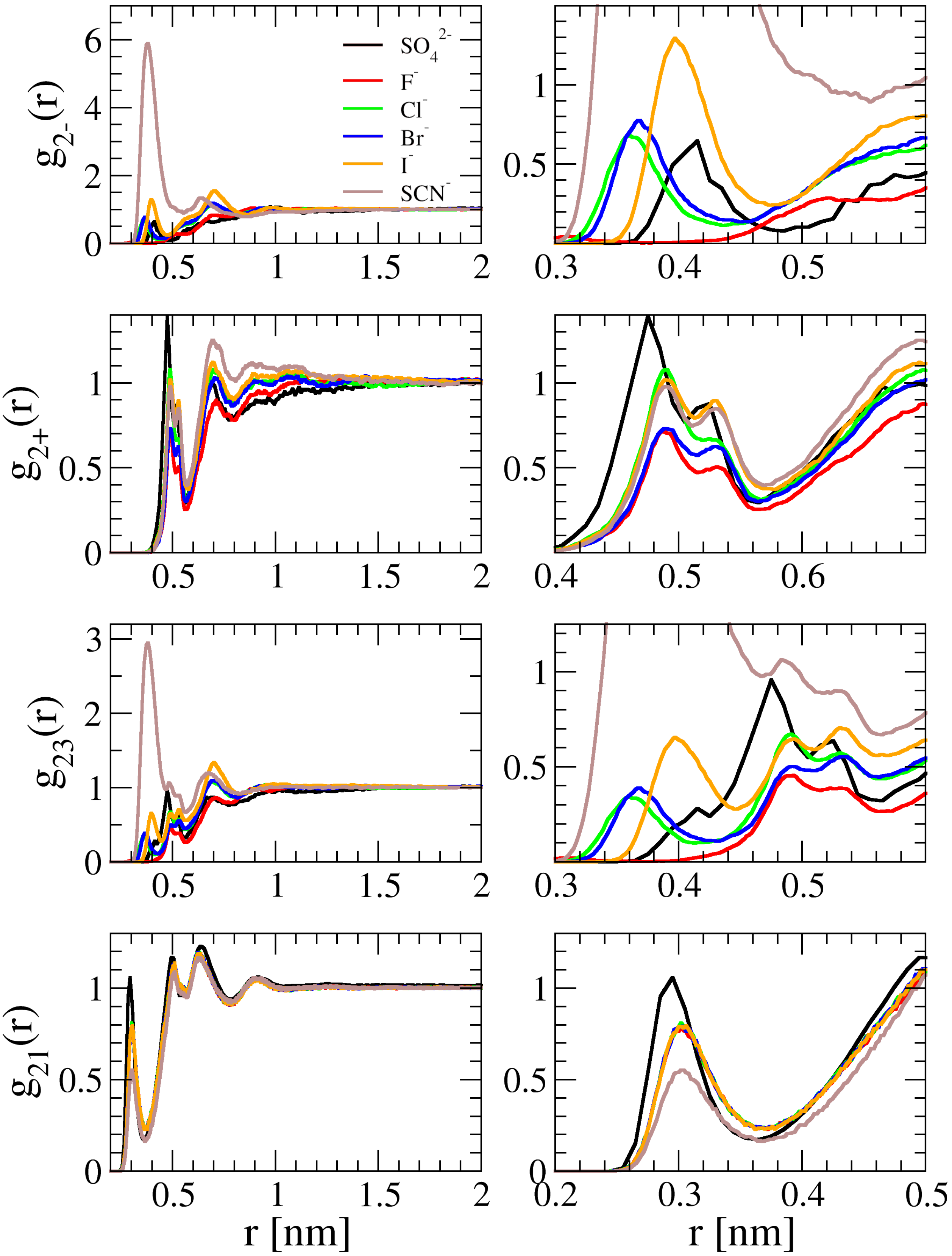

Figure S1: Left panels present radial distribution functions of anions ($g_{2-}(r)$), sodium cation ($g_{2+}(r)$), net salt ($g_{23}(r)$), and water ($g_{21}(r)$) around center of mass of the caffeine molecule. Right panels present the zoom into first peak of respective RDFs on the left to better distinguish affinity of individual salts, ions or water. All simulations were performed with single caffeine molecule in 1 M salt solution. In case of sulfate and thiocyanate the distance is measured from the sulfur atom, oxygen atom was used for water. Color coding of salts and their ions, respectively, follows the legend.

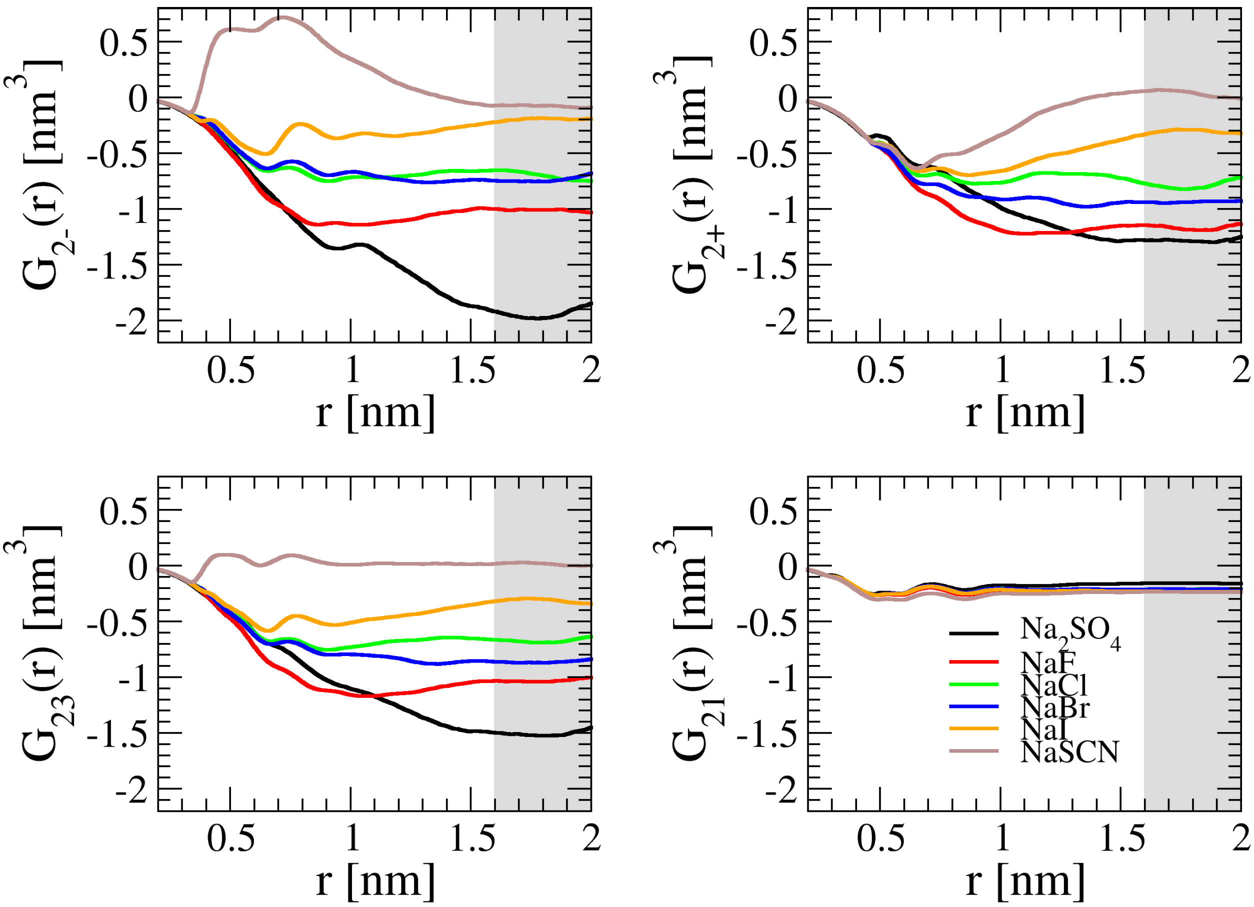

Figure S2: Kirkwood-Buff integrals (KBI) evaluated from radial distribution functions in Figure S1. The KBI of caffeine-anion (top left, $G_{2-}(r)$), caffeine-sodium (top right, $G_{2+}(r)$), caffeine-salt (bottom left, $G_{23}(r)$), and caffeine-water (bottom right, $G_{21}(r)$). The thermodynamic value of KBIs corresponds to the plateau value at large r, here indicated by the shadowed interval 1.6–2 nm.

S2 Determination of partial proximal distribution function

Caffeine molecule possesses complex surface, which is a combination of functional group of varying polarity and hydrophobicity. In order to determine rational salt-affinities to caffeine molecule, we have calculated proximal distribution functions of water and ions. We have partitioned caffeine surface to functional groups, and calculated partial proximal distribution functions, in order to reflect local hydration and salt affinities. The $g_{\mathrm{prox,Ij}}(r_{Ij})$ of atoms j from the functional group I is defined as

$$g_{\mathrm{prox,Ij}}(r_{Ij}) = \frac{\rho_j(r_{Ij})}{\rho_j^0} = \frac{1}{\rho_j^0}\frac{\Delta N_j(r_{Ij})}{\Delta V(r_{Ij})}$$

where the $\Delta N_j(r_{Ij}) = N_j(r_{Ij} + \Delta r) - N_j(r_{Ij})$ is the number of particles j in the proximal volume shell around group I (of thickness Δr) at distance r_{ij}, the $\Delta V(r_{Ij}) = V(r_{Ij} + \Delta r) - V(r_{Ij})$ is a volume of this shell, and ρ_j^0 is the bulk density of particles j.

A practical realization of $g_{\mathrm{prox,Ij}}(r_{Ij})$ calculation is presented in Figure S3. In order to properly normalize $g_{\mathrm{prox,Ij}}(r_{Ij})$ proximal volumes, or rather, proximal volume layers $\Delta V(r_{Ij})$, have to be determined (see the inset in Figure S3, colored consistently with the legend). Knowledge of $\Delta V(r_{Ij})$ is also required to quantify the contribution (weight) of individual functional groups in the overall (net) proximal distribution function g_{prox}.

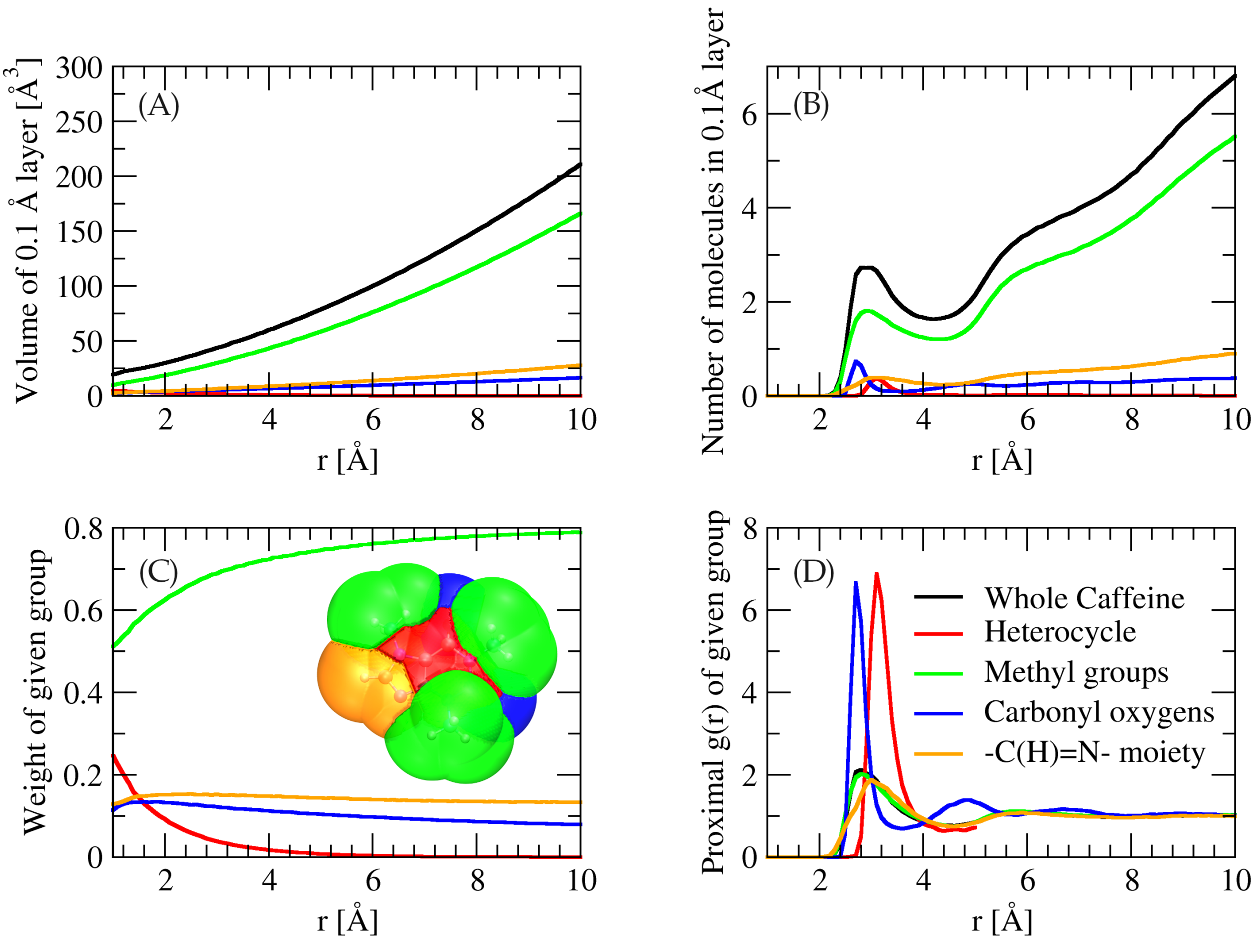

Figure S3: Illustration of calculation of partial proximal distribution functions on the example of water oxygen around functional groups (I) of caffeine. The procedure involves the determination of (a) proximal volume layer, (b) proximal coordination number, (c) statistical weights of functional groups I. Resulting partial proximal distribution functions, shown in (d), present deeper insight in the local hydration. The advantages of g_{prox} were discussed elsewhere [4]

.

S3 Calibration of coarse grained model of caffeine

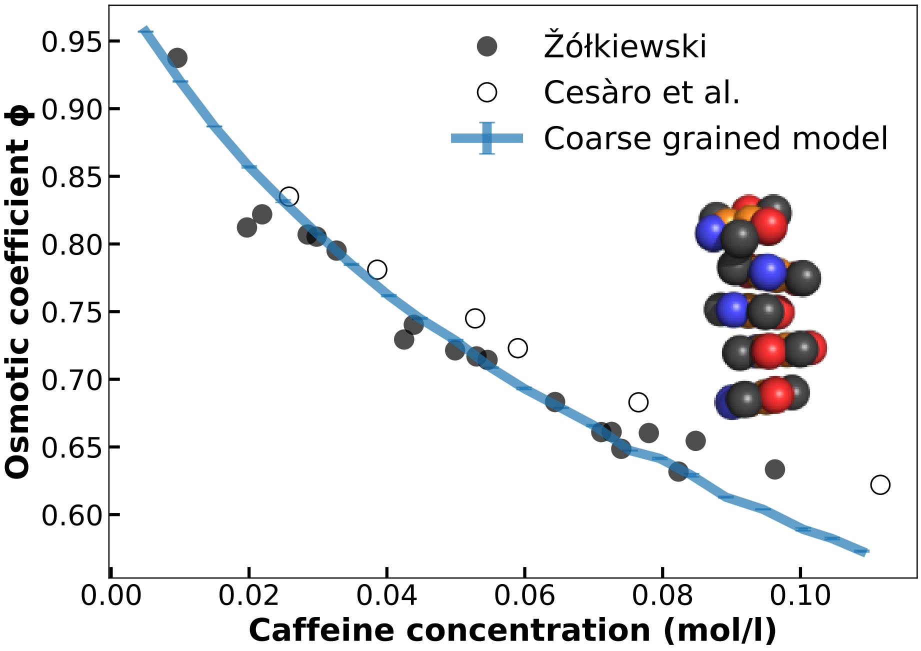

Figure S4: *Left* Osmotic coefficient for caffeine in water as a function of caffeine concentration observed experimentally (circles) by Žółkiewski [5](25 °C) and Cesàro *et al.* [6] (30 °C), and from coarse grained MC simulations at 25 °C (line). Insert shows a representable configuration observed from the coarse grained MC simulations at 0.1 mol/l, illustrating face-to-face stacking of caffeine.

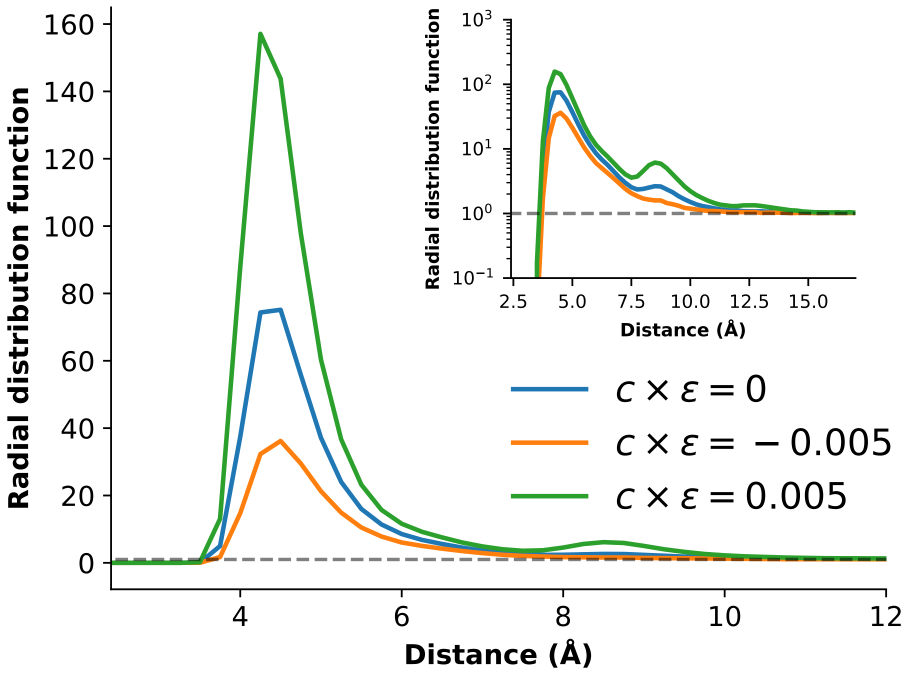

Figure S5: Radial distribution function for caffeine-caffeine (molarity of caffeine is 12.5 mM) in the absence ($c \times \epsilon = 0$) and in the presence of salts. The inset present y-axis in the log scale, highlighting the locations of stacked caffeine molecules in 1st, 2nd and 3rd layer (see the snapshot in Figure S4), but also the decay of RDF to 1. We note that the corresponding Kirkwood-Buff integral, $G_{22} \approx 20 \cdot 10^3$ Å^3, agrees well with our analysis (Figure S9) and experimental data in the literature [5].

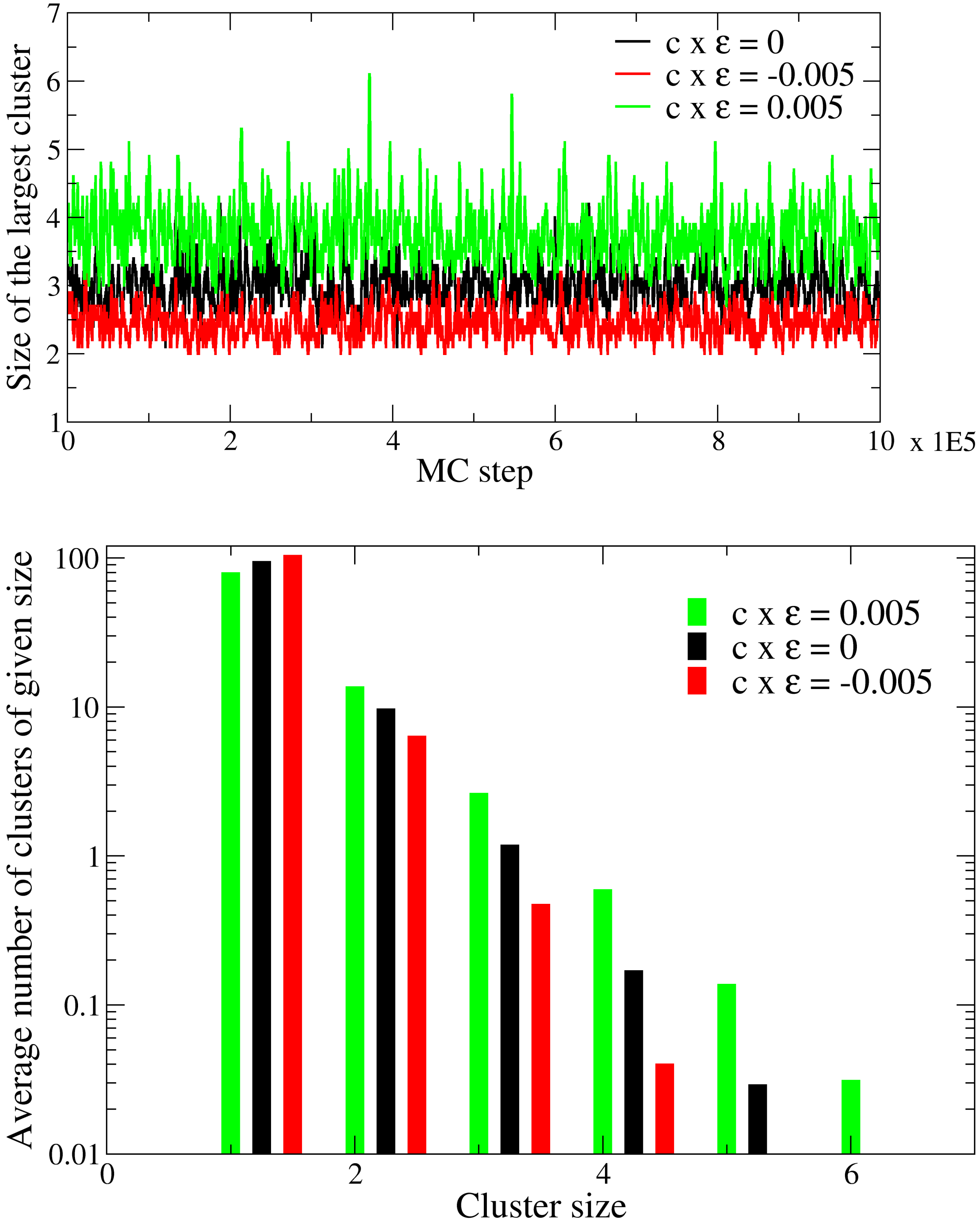

Figure S6: Cluster analysis of caffeine in neat water and salt solutions from the coarse-grained Monte Carlo simulations. The concentration of caffeine is 12.5 mM and salt was present via the product $c \times \epsilon$ (*cf.* 10) taking the value of either 0.005 (*salting-out*, green), -0.005 (*salting-in*, red), or 0 (absence of salt, black). The distance cutoff for the cluster analysis was set to the maximum of the first peak in RDF (solvation shell) in Fig. S5, $r_{\text{cut}} = 5$ Å *Top.* Running average of the size (the window is 10 sample points) of the largest cluster of caffeine molecules throughout the trajectory. *Bottom* Cluster distribution of caffeine averaged over the trajectories in the absence and presence of salt. The distribution is normalized such that $\sum_i y_i \times x_i = 128$, *i.e.*, the total number of caffeine molecules in the system.

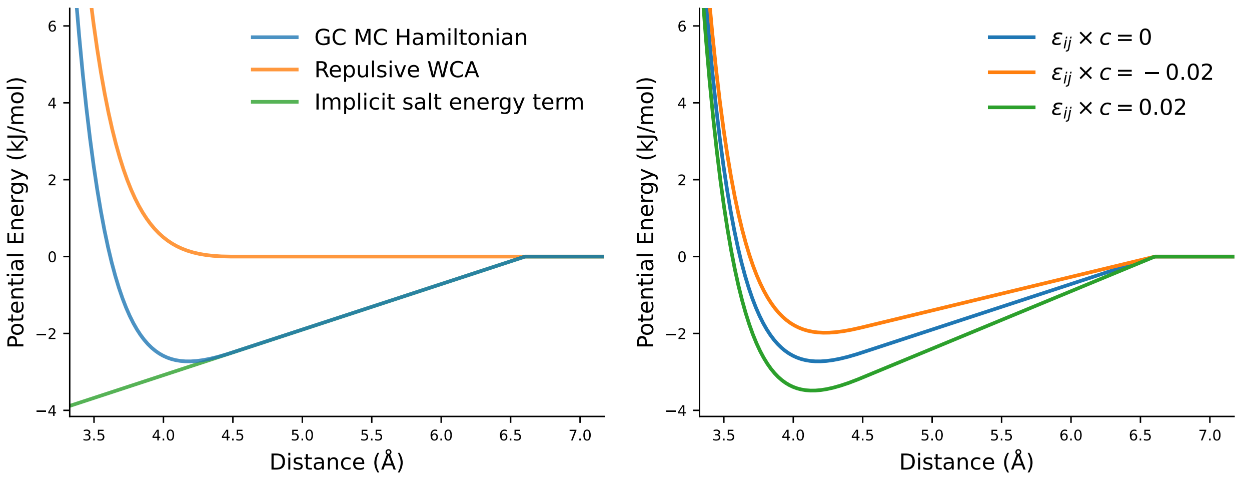

Figure S7: Illustration of the GC MC Hamiltonian employed in this work as a function of distance. *Left.* Decomposition of the GC MC Hamiltonian into the contributions from the WCA (first second of Eq. 10 and the implicit salt energy (second term of Eq. 10). It may be noticed that WCA potential accounts for Pauli-repulsion with the potential becoming zero for distances equal or greater than $2^{1/6}\sigma$. The implicit salt energy term is effectively a linear function with increasing particle-particle separation with the term reaching maximum potential energy at full separation of the two spheres. The point of full separation distance can be identified by the point of the cusp/spinode that is having the characteristic distance $(\sigma_1 + \sigma_2)/2 + 2 * \sigma_{\text{probe}}$. The parameters utilized are: $\sigma_{ij} = 4.0$ Å, $\sigma_{\text{probe}} = 1.3$ Å, $\gamma_{ij} = 0.0572$ kJ/mol/Å^2, $\epsilon_{ij,\text{WCA}} = 0.5$ kJ/mol, $\varepsilon_{ij} \times c = 0$. *Right.* Addition of salt effects to the potential though the product $\varepsilon_{ij} \times c$ [kJ/mol/Å^2]. It can be seen that positive values for the product causes the potential to be more attractive while negative values causes for the product causes the potential to be less attractive. The parameters utilized are the same the left plot with the exception of the product which is color-coded in accordance to the legend.

S4 Alternative coarse grained model calibration

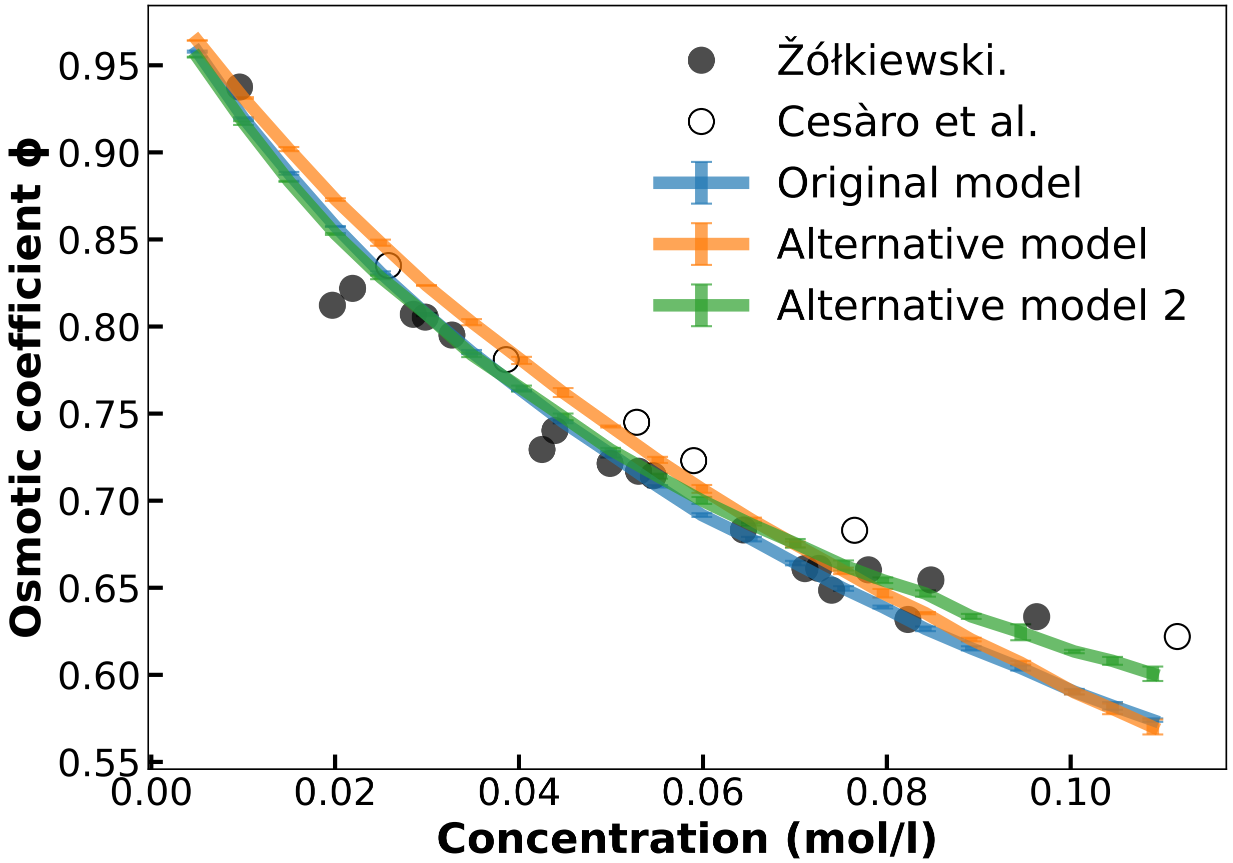

Figure S8: Osmotic coefficient for caffeine in water as a function of caffeine concentration observed experimentally (circles) by Žółkiewski[5](25 °C) and Cesàro et al.[6] (30 °C), and from coarse grained MC simulations at 25 °C (lines). The original model presented in the manuscript differs from the alternative models by the introduction of positive tension (0.0100, orange) and negative tension (-0.0200, green) on the methyl groups. In response to the introduction of attraction and repulsion on the methyl groups the tension on the aromatic spheres decreased to 0.0480 for positive tension on the methyl spheres and increased to 0.0660 for negative tension on the methyl spheres. The effect of repulsion/attraction on the methyl groups (and most likely the polar groups) mainly determines the curvature of the osmotic coefficient at high concentrations, while a too high tension would also cause a full aggregation, characterized by a discontinuity in the osmotic coefficient (data not shown).

S5 Complete Kirkwood-Buff analysis of MC-SASA simulation data

The parameters in Table 3 determine the activity and volumetric properties of the caffeine-salt solution. Employing KB-inversion procedure via Eq. (11), (12), all six KB-integrals were determined. Figures S9 and S10 present KB-integrals for series of caffeine concentrations as a function of salt concentration. The consequences of salt-caffeine interactions, which are implicitly accounted for in MC-SASA model are best manifested in KB-integrals, which include caffeine species, *i.e.*, G_{12}, G_{22}, and G_{23}. These are compared in Figure S11 (mirrored for clarity, $\underline{m}_3 = 0\,\mathrm{mol/kg}$ in the middle is increasing to $\underline{m}_3 = 0.5\,\mathrm{mol/kg}$ on the sides) for the salting-in (TFE = −0.01) and salting-out (TFE = 0.01) salts, respectively. Clearly, the two qualitatively opposite salt actions can be distinguished in every KB-integral, which are described in more detail below.

While the caffeine-water (G_{12}) is weakly increasing with the addition of salting-in salt (left panel), it is decreasing in the presence of salting-out salt (right panel). The salt effect magnifies at higher caffeine concentrations.

The caffeine-caffeine interaction (G_{22}), which roughly represents the caffeine self-association, is weakened by salting-in salt approximately to the level of highly diluted caffeine in neat water. As expected, the salting-out salt intensifies the caffeine self association to values that are by factor 2-3 higher than in neat water.

The last row compares caffeine-salt interaction (G_{23}). As expected, in case of salting-in salt the KB-integral is positive, i.e., salt in enriched in the vicinity of caffeine, while the salting-out salt is depleted, represented by a large negative KB-integral. Salt enrichment or depletion strengthen with increasing caffeine concentration, and while the concentration of salting-in salt has only minor effect on G_{23}, the salting-out salt decreases G_{23} rather markedly.

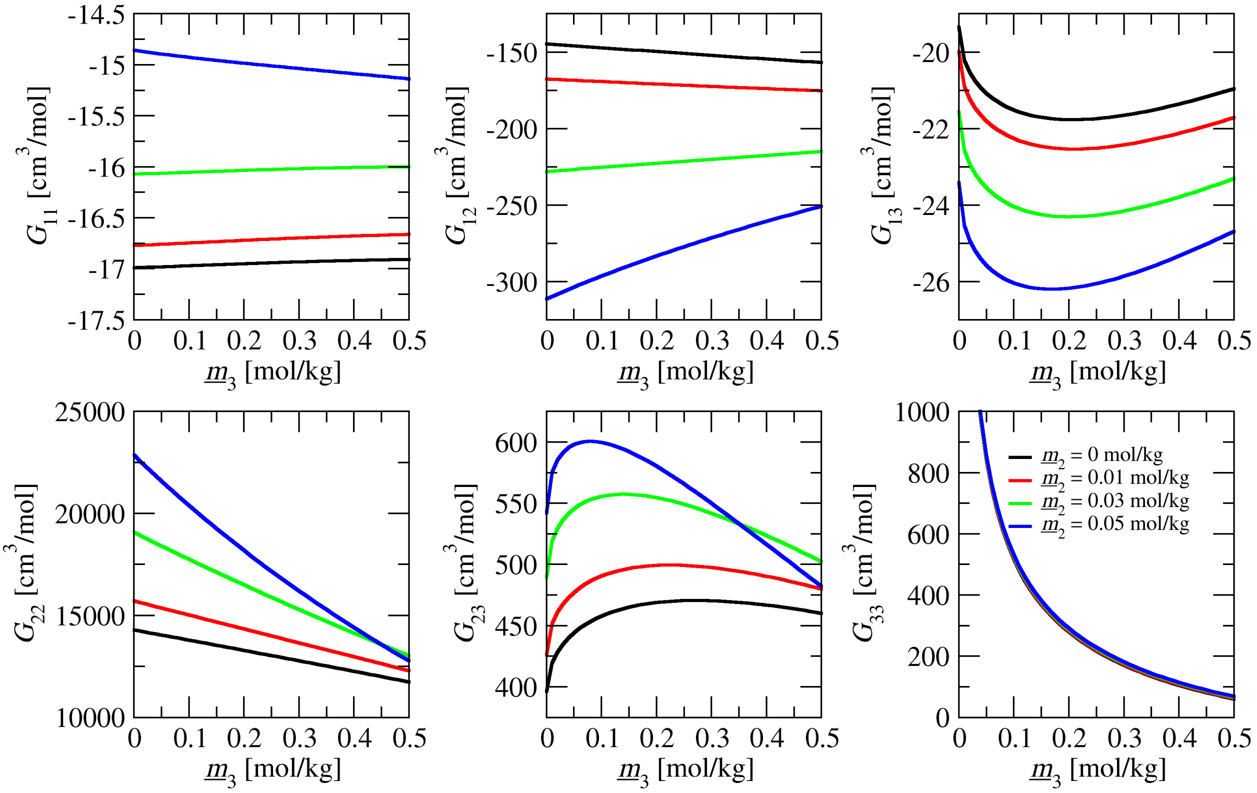

Figure S9: Complete set of Kirkwood-Buff integrals for TFE = -0.01 for selected caffeine concentrations (0-0.05 mol/kg) up to the salt concentration $\underline{m}_3 = 0.5\,\mathrm{mol/kg}$.

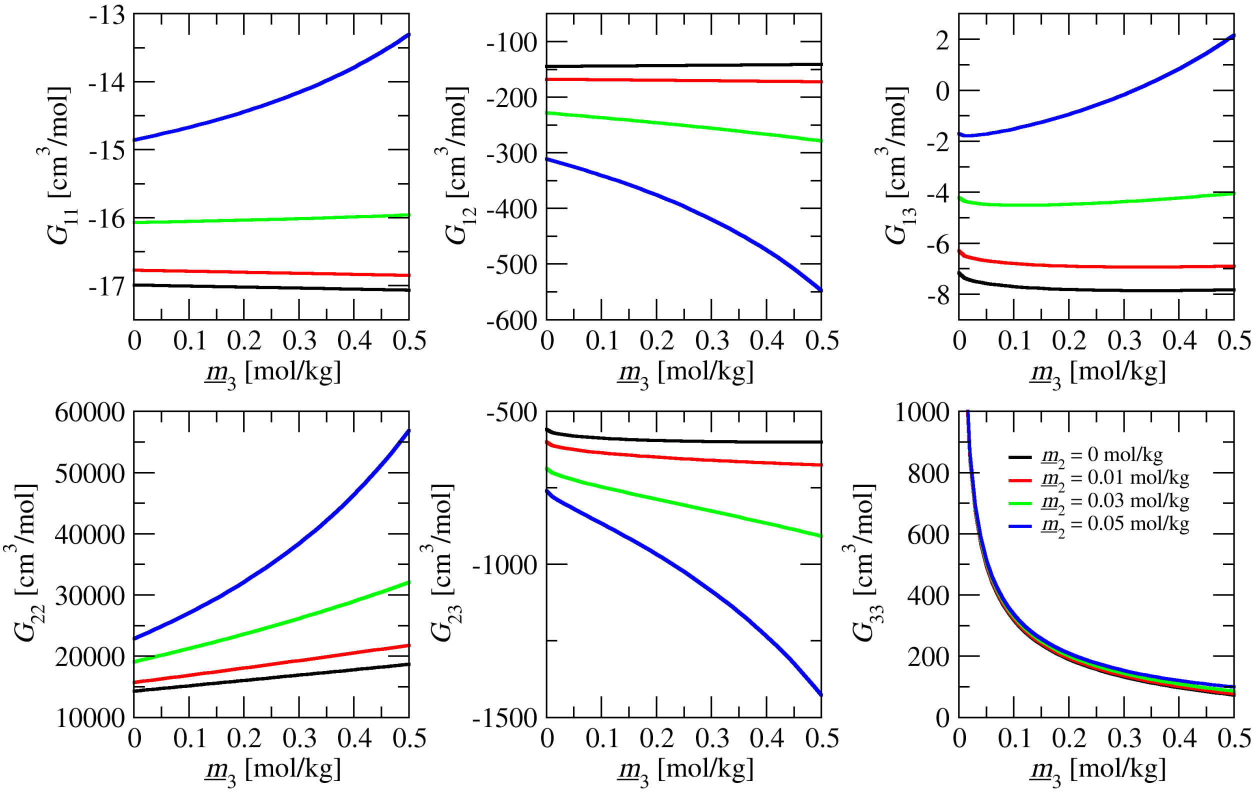

Figure S10: Complete set of Kirkwood-Buff integrals for TFE = 0.01 for selected caffeine concentrations (0-0.05 mol/kg) up to the salt concentration $\underline{m}_3 = 0.5$ mol/kg.

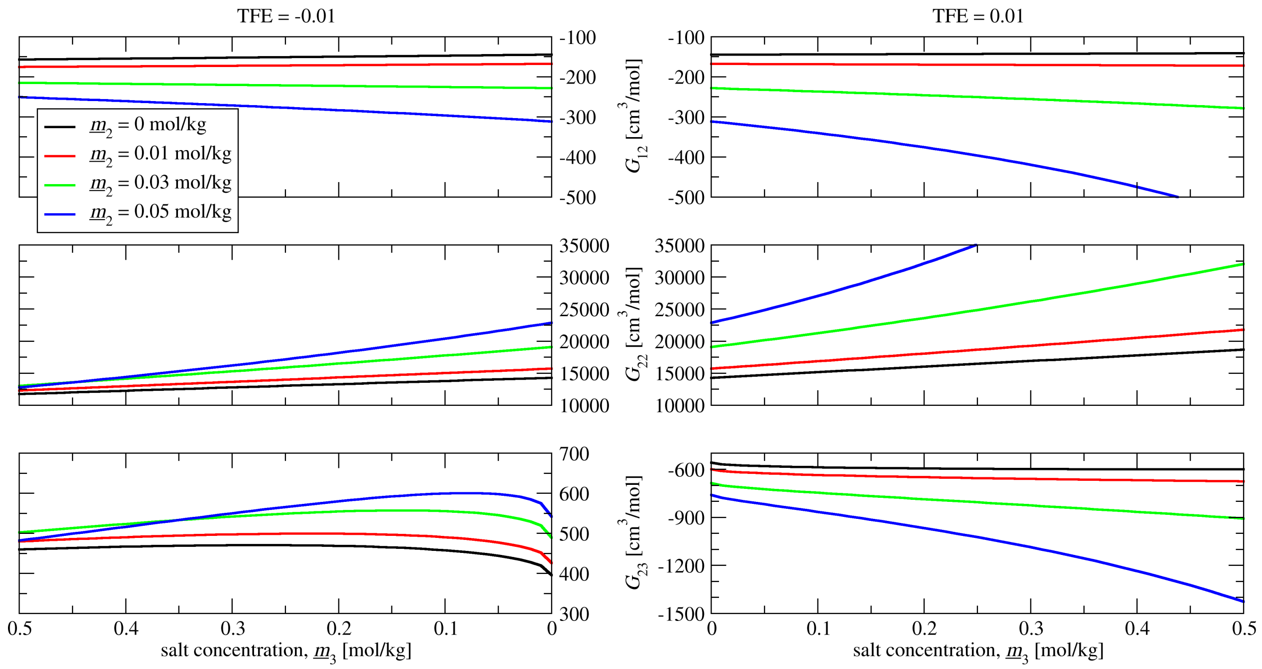

Figure S11: The most prominent (caffeine involved) Kirkwood-Buff integrals for TFE = -0.01 (left panels), and TFE = 0.01 (right panels) as obtained via Kirkwood-Buff inversion (Eq. (11), (12)) of the MC-SASA simulation data presented in Table S4, which were fitted and are represented by parameters in Table 3. Color of the line represent caffeine concentration (see the legend). 0 mol/kg stays for infinite dilution of caffeine.

S6 Higher-order terms in fitting of ΔOsm data

Some of ΔOsm data points (circles) in Fig. 2 deviate from the linear trend line, which is expected if

$$\frac{\Delta \mathrm{Osm}}{\underline{m}_2 \cdot \underline{m}_3} = k_\mathrm{s} \approx \frac{1}{RT}\left(\frac{\partial \mu_2}{\partial \underline{m}_3}\right)_{\underline{m}_2, \mu_1, T}$$

This indicates that additional terms in the expansion of $\frac{\Delta \mathrm{Osm}}{\underline{m}_2 \cdot \underline{m}_3}$, i.e., dependence in $\underline{m}_2$ and/or $\underline{m}_3$ is needed. In the manuscript, we assigned this deviation to the caffeine concentration ($\underline{m}_2$), which resulted in Eq. (3). The protocol of k_s determination is illustrated on the example of NaCl salt, see Fig. S12(A).

The analysis of slopes in Fig. S12(C) shows that the dependence of $\frac{\Delta \mathrm{Osm}}{\underline{m}_2 \cdot \underline{m}_3}$ in the salt concentration ($\underline{m}_2$) is significantly smaller than in the caffeine concentration (see slopes in Fig. S12(B)), and thus can be neglected in our ΔOsm model (Eq. (3)).

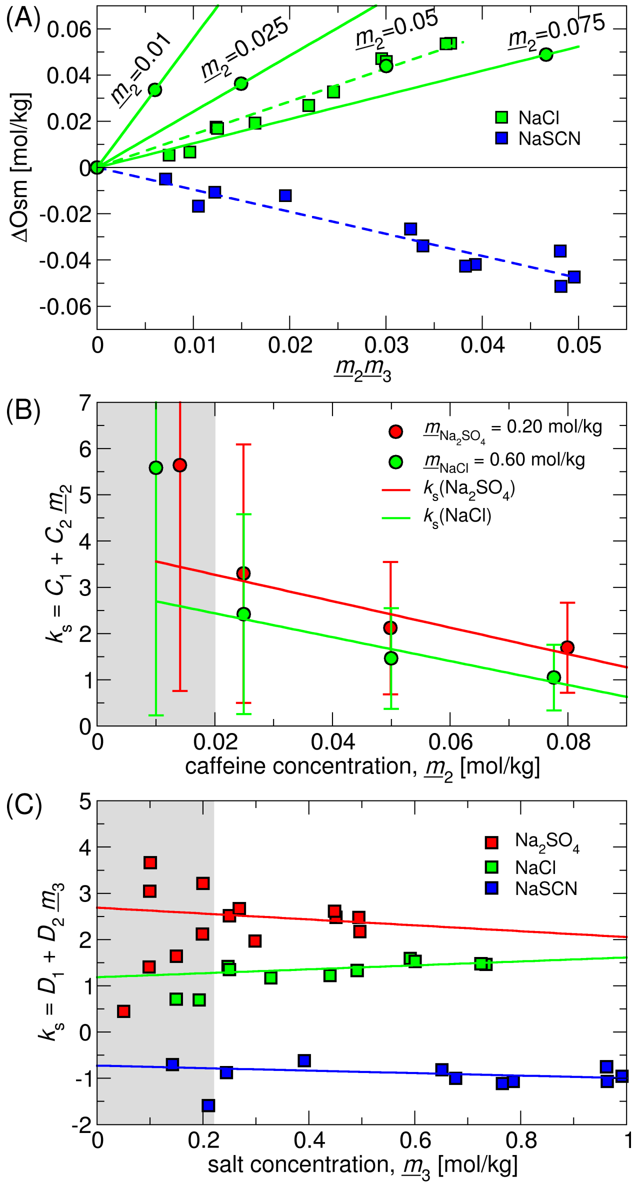

Figure S12: (A) Excess osmolality, ΔOsm, from VPO measurements of caffeine-salt solutions: NaCl (green), and NaSCN (blue). Measurements at constant salt concentration, NaCl (0.6 mol/kg) and varying caffeine concentration are shown with circles. Linear regression (full line) of individual points illustrates the influence of caffeine concentration ($\underline{m}_2$) on salting out constant (k_s), which is shown in (B). Data for sulfate are shown in Fig. 2. Data gathered at constant caffeine concentration $\underline{m}_2 = 50$ mmol/kg and varying salt concentrations are presented in squares with linear regression indicated by dashed lines. Deviation of data from this slope illustrate the influence of salt concentration ($\underline{m}_3$) on salting out constant (k_s), which is shown in (C). In panels (B) and (C) the lowest caffeine or salt concentrations (gray domain) were omitted from the fitting.

S7 VPO – raw experimental data

Table S2: Experimental ternary and calculated binary osmolalities for constant caffeine concentration ($\underline{m}_2$ = 0.05 mol/kg) determined at 37°C used in eq. (2) to calculate ΔOsm.

$\underline{m}_2$	$\underline{m}_3$	Osm($\underline{m}_2, \underline{m}_3$)	Osm($\underline{m}_2$, 0)	Osm(0, $\underline{m}_3$)
Na_2SO_4				
0.0500	0.0499	175	42	132
0.0496	0.0987	297	42	248
0.0499	0.0994	307	42	250
0.0500	0.1002	312	42	251
0.0500	0.1496	417	42	362
0.0499	0.1994	533	42	470
0.0500	0.1998	545	42	471
0.0499	0.2501	649	42	575
0.0500	0.2684	691	42	613
0.0496	0.3004	749	42	678
0.0500	0.4487	1069	42	968
0.0500	0.4516	1072	42	974
0.0499	0.4948	1160	42	1056
0.0496	0.5004	1163	42	1067
NaCl				
$\underline{m}_2$	$\underline{m}_3$	Osm($\underline{m}_2, \underline{m}_3$)	Osm($\underline{m}_2$, 0)	Osm(0, $\underline{m}_3$)
0.0499	0.1495	331	42	279
0.0499	0.1930	413	42	358
0.0496	0.2490	527	42	461
0.0500	0.2497	528	42	462
0.0499	0.3285	675	42	606
0.0499	0.4404	888	42	812
0.0496	0.4951	994	42	913
0.0499	0.5921	1187	42	1094
0.0500	0.6009	1202	42	1111
0.0499	0.7356	1459	42	1366
0.0485	0.7476	1480	41	1389
NaSCN				
$\underline{m}_2$	$\underline{m}_3$	Osm($\underline{m}_2, \underline{m}_3$)	Osm($\underline{m}_2$, 0)	Osm(0, $\underline{m}_3$)
0.0500	0.1422	298	42	261
0.0500	0.2101	407	42	381
0.0499	0.2446	474	42	442
0.0500	0.3910	733	42	703
0.0500	0.5871	1054	42	1058
0.0500	0.6517	1194	42	1178
0.0500	0.6775	1235	42	1227
0.0500	0.7660	1394	42	1394
0.0500	0.7865	1434	42	1434
0.0500	0.9633	1771	42	1780
0.0500	0.9616	1783	42	1777
0.0500	0.9922	1833	42	1838

Table S3: Experimental ternary and calculated binary osmolalities for constant salt ($\underline{m}_3$) and varying caffeine ($\underline{m}_2$) concentrations determined at 37°C used in eq. (2) to calculate ΔOsm.

Na_2SO_4				
$\underline{m}_2$	$\underline{m}_3$	$Osm(\underline{m}_2, \underline{m}_3)$	$Osm(\underline{m}_2, 0)$	$Osm(0, \underline{m}_3)$
0.0141	0.1994	499	13	470
0.0249	0.1994	509	23	470
0.0499	0.1994	533	42	470
0.0750	0.1994	553	58	470
NaCl				
$\underline{m}_2$	$\underline{m}_3$	$Osm(\underline{m}_2, \underline{m}_3)$	$Osm(\underline{m}_2, 0)$	$Osm(0, \underline{m}_3)$
0.0100	0.2497	495	10	469
0.0250	0.2497	509	23	469
0.0500	0.2497	528	42	469
0.0745	0.2497	546	58	469
0.0100	0.6009	1157	10	1114
0.0249	0.6009	1173	23	1114
0.0500	0.6009	1200	42	1114
0.0776	0.6009	1222	59	1114

S8 Excess chemical potential of caffeine μ_2^{ex} as determined in MC-SASA simulations

Table S4: Caffeine excess chemical potential as calculated by coarse-grained MC-model for various caffeine ($\underline{m}_2$) and salt ($\underline{m}_3$) concentrations. These data are used in KB-inversion analysis. We remind that TFE = 0.01 represents a salting out salt, and TFE = −0.01 represents a salting in salt.

$\underline{m}_2$	$\underline{m}_3$	TFE	μ_2^{ex}
0.1	0	0.01	-1.104704707
0.1	0.5	0.01	-1.063771708
0.1	1	0.01	-1.080893439
0.05	0	0.01	-0.640133749
0.05	0.5	0.01	-0.439448796
0.05	1	0.01	-0.363003062
0.025	0	0.01	-0.374670641
0.025	0.5	0.01	-0.007543382
0.025	1	0.01	0.253952198
0.0125	0	0.01	-0.193591533
0.0125	0.5	0.01	0.238553423
0.0125	1	0.01	0.604889557
0.1	0	-0.01	-1.133344759
0.1	0.5	-0.01	-1.461232586
0.1	1	-0.01	-1.621435432
0.05	0	-0.01	-0.637898649
0.05	0.5	-0.01	-0.997269911
0.05	1	-0.01	-1.447948748
0.025	0	-0.01	-0.352748088
0.025	0.5	-0.01	-0.841550012
0.025	1	-0.01	-1.354229464
0.0125	0	-0.01	-0.205080942
0.0125	0.5	-0.01	-0.731491217
0.0125	1	-0.01	-1.301941533

References

[1] B. A. Rogers, T. S. Thompson, and Y. Zhang, "Hofmeister anion effects on thermodynamics of caffeine partitioning between aqueous and cyclohexane phases," *The Journal of Physical Chemistry B*, vol. 120, pp. 12596–12603, Dec. 2016.

[2] A. Al-Maaieh and D. R. Flanagan, "Salt Effects On Caffeine Solubility, Distribution, And Self-Association," *Journal of Pharmaceutical Sciences*, vol. 91, pp. 1000–1008, apr 2002.

[3] M. B. Gee, N. R. Cox, Y. Jiao, N. Bentenitis, S. Weerasinghe, and P. E. Smith, "A Kirkwood-Buff Derived Force Field for Aqueous Alkali Halides," *Journal of Chemical Theory and Computation*, vol. 7, pp. 1369–1380, may 2011.

[4] B. Lin and B. M. Pettitt, "Note: On the Universality of Proximal Radial Distribution Functions of Proteins," *J. Chem. Phys.*, vol. 134, no. 10, pp. 2011–2013, 2011.

[5] M. Žółkiewski, "Kirkwood-buff integrals and density fluctuations in aqueous solution of caffeine," *Journal of Solution Chemistry*, vol. 16, pp. 1025–1034, Dec. 1987.

[6] A. Cesaro, E. Russo, and V. Crescenzi, "Thermodynamics of caffeine aqueous solutions," *The Journal of Physical Chemistry*, vol. 80, pp. 335–339, Jan. 1976.
